# Supplementary material for: How effective was England's teenage pregnancy strategy? A comparative analysis of high-income countries
Source: Soc Sci Med. 2021 Feb;270:113685. doi: 10.1016/j.socscimed.2021.113685 (PMC7895815; doi:10.1016/j.socscimed.2021.113685)

# Data sources and code used in analyses

## Data sources

| Data used | Source | url |
| --- | --- | --- |
| Numbers of abortions to under-20s in Europe | European Health Information Gateway - Health for all explorer | <https://gateway.euro.who.int/en/hfa-explorer/> |
| Populations of countries by age | Human mortality database | <https://www.mortality.org> |
| Birth totals of countries by age | Human fertility database | <https://www.humanfertility.org> |
| Numbers of births by age, Scotland | NRS Scotland | <https://www.nrscotland.gov.uk/statistics-and-data/statistics/stats-at-a-glance/registrar-generals-annual-review/2017> |
| Number of conceptions by age, Scotland 1994-2016 | ISD Scotland | <http://www.isdscotland.org/Health-Topics/Sexual-Health/Publications/2018-07-03/mat_tp_table1.xls> |
| Estimates of yearly gross domestic product (GDP), mobile phone ownership, proportion of females in population, proportion of population resident in urban settings | World Bank Open Data | <https://data.worldbank.org> |
| Conceptions in England and Wales to under-20s | Office for National Statistics | <https://www.ons.gov.uk/peoplepopulationandcommunity/birthsdeathsandmarriages/conceptionandfertilityrates/bulletins/conceptionstatistics/2017> |
| Pregnancy and birth rates to teenagers in the USA | The Guttmacher Institute | <https://www.guttmacher.org/report/us-adolescent-pregnancy-trends-2013> |
| New Zealand births by age of mother | Statistics New Zealand | <http://stats.govt.nz> |
| New Zealand abortions by age of mother | Statistics New Zealand | <http://stats.govt.nz> |
| Public spending on education | OECD Data | <https://data.oecd.org/eduresource/public-spending-on-education.htm> |

## Project code repositories

All R code used to run analyses and produce visualisations is available online. The interactive Shiny app is also published online and can be used to repeat ITS analyses without prior installation of R.

Code used to run the analyses and produce the graphs is available online in two Github repositories:

- [[link anonymised]](https://github.com/andrewbaxter439/teen-preg-project) - all data importing and tidying and code to run Synthetic Control analyses. (archived version at [doi.org/10.5281/ZENODO.3822193](https://doi.org/10.5281/ZENODO.3822193))
- [[link anonymised]](https://github.com/andrewbaxter439/ITS_shinyapp) - code for the Shiny app to run Interrupted Time Series analyses (archived version at [doi.org/10.5281/ZENODO.3822198](https://doi.org/10.5281/ZENODO.3822198))

The ITS Shiny app is also published at [[link anonymised]](https://andybaxter.shinyapps.io/teen_preg_uk_its/) and can be run without installation of R or experience using R.

## Packages used in R

- Shiny (Chang, Cheng, Allaire, Xie, & McPherson, 2019)
- nlme (Pinheiro, Bates, DebRoy, Sarkar, & R Core Team, 2019)
- tidyverse (Wickham, 2017)
- svglite (Wickham, Henry, Luciani, Decorde, & Lise, 2019)
- broom (Robinson & Hayes, 2020)
- car (Fox & Weisberg, 2019)
- XLConnect (Mirai Solutions GmbH, 2018)
- officer (Gohel, 2020a)
- rvg (Gohel, 2020b)
- patchwork (Pedersen, 2019)
- export (Wenseleers & Vanderaa, 2018)
- gganimate (Pedersen & Robinson, 2019)
- readxl (Wickham & Bryan, 2019)
- Synth (Abadie, Diamond, & Hainmueller, 2011)
- SPHSUgraphs (Baxter, 2019)
- ggrepel (Slowikowski, 2020)
- ggpubr (Kassambara, 2020)
- gtools (Warnes, Bolker, & Lumley, 2020)
- foreach (Microsoft & Weston, 2020)
- doParallel (Corporation & Weston, 2019)
- plotly (Sievert, 2018)
- magrittr (Bache & Wickham, 2014)

Abadie, A., Diamond, A., & Hainmueller, J. (2011). Synth: An R Package for Synthetic Control Methods in Comparative Case Studies. *Journal of Statistical Software*, *42*(13), 1–17. Retrieved from http://www.jstatsoft.org/v42/i13/

Bache, S. M., & Wickham, H. (2014). *magrittr: A Forward-Pipe Operator for R*. Retrieved from https://cran.r-project.org/package=magrittr

Baxter, A. J. (2019). *SPHSUgraphs: Select SPHSU Brand Colours For ggplot2 Graphs*. Retrieved from https://github.com/andrewbaxter439/SPHSUgraphs

Chang, W., Cheng, J., Allaire, J. J., Xie, Y., & McPherson, J. (2019). *Shiny: Web Application Framework for R*. Retrieved from https://cran.r-project.org/package=shiny

Corporation, M., & Weston, S. (2019). *doParallel: Foreach Parallel Adaptor for the “parallel” Package*. Retrieved from https://cran.r-project.org/package=doParallel

Fox, J., & Weisberg, S. (2019). *car: An R Companion to Applied Regression* (Third). Retrieved from https://socialsciences.mcmaster.ca/jfox/Books/Companion/

Gohel, D. (2020a). *officer: Manipulation of Microsoft Word and PowerPoint Documents*. Retrieved from https://cran.r-project.org/package=officer

Gohel, D. (2020b). *rvg: R Graphics Devices for Vector Graphics Output*. Retrieved from https://cran.r-project.org/package=rvg

Kassambara, A. (2020). *ggpubr: “ggplot2” Based Publication Ready Plots*. Retrieved from https://cran.r-project.org/package=ggpubr

Microsoft, & Weston, S. (2020). *foreach: Provides Foreach Looping Construct*. Retrieved from https://cran.r-project.org/package=foreach

Mirai Solutions GmbH. (2018). *XLConnect: Excel Connector for R*. Retrieved from https://cran.r-project.org/package=XLConnect

Pedersen, T. L. (2019). *patchwork: The Composer of Plots*. Retrieved from https://cran.r-project.org/package=patchwork

Pedersen, T. L., & Robinson, D. (2019). *gganimate: A Grammar of Animated Graphics*. Retrieved from https://cran.r-project.org/package=gganimate

Pinheiro, J., Bates, D., DebRoy, S., Sarkar, D., & R Core Team. (2019). *nlme: Linear and Nonlinear Mixed Effects Models*. Retrieved from https://cran.r-project.org/package=nlme

Robinson, D., & Hayes, A. (2020). *broom: Convert Statistical Analysis Objects into Tidy Tibbles*. Retrieved from https://cran.r-project.org/package=broom

Sievert, C. (2018). *plotly: Plotly for R*. Retrieved from https://plotly-r.com

Slowikowski, K. (2020). *ggrepel: Automatically Position Non-Overlapping Text Labels with “ggplot2.”* Retrieved from https://cran.r-project.org/package=ggrepel

Warnes, G. R., Bolker, B., & Lumley, T. (2020). *gtools: Various R Programming Tools*. Retrieved from https://cran.r-project.org/package=gtools

Wenseleers, T., & Vanderaa, C. (2018). *export: Streamlined Export of Graphs and Data Tables*. Retrieved from https://cran.r-project.org/package=export

Wickham, H. (2017). *tidyverse: Easily Install and Load the “Tidyverse.”* Retrieved from https://cran.r-project.org/package=tidyverse

Wickham, H., & Bryan, J. (2019). *readxl: Read Excel Files*. Retrieved from https://cran.r-project.org/package=readxl

Wickham, H., Henry, L., Luciani, T. J., Decorde, M., & Lise, V. (2019). *svglite: An “SVG” Graphics Device*. Retrieved from https://cran.r-project.org/package=svglite

# Interrupted Time Series analysis outputs

Outputted from [[link anonymised]](https://andybaxter.shinyapps.io/teen_preg_uk_its/)

## All pregnancy rates across England, Scotland and Wales


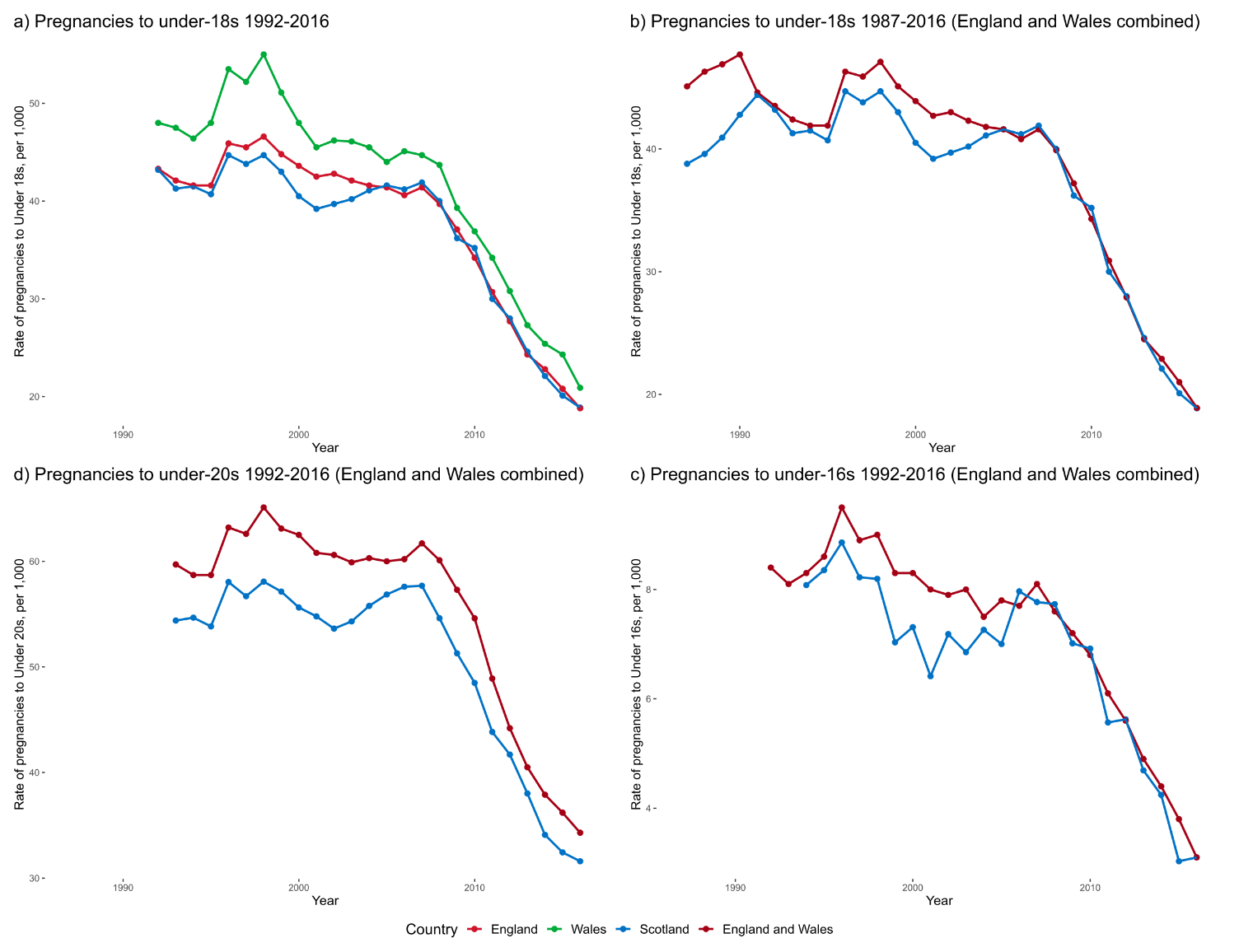


England saw a 60% drop in pregnancies between 1998 and 2016, from 46.6 to 18.8 pregnancies per 1,000 women. Scotland rates fell by 58% (from 44.7 to 18.9 pregnancies per 1,000 women) and Wales by 62% (from 55.0 to 20.9 pregnancies per 1,000 women). England and Wales combined showed decreases in pregnancies amongst under-16 and under-20-year-olds of 66% and 47% respectively, with Scotland seeing similar drops of 62% and 46%. When comparing England under-18 pregnancy rates with aggregated England and Wales rates, we found very little difference between the two, with an average prediction error of 0.25 pregnancies per 1,000 women across all observed years.

## England 1992 - 2016


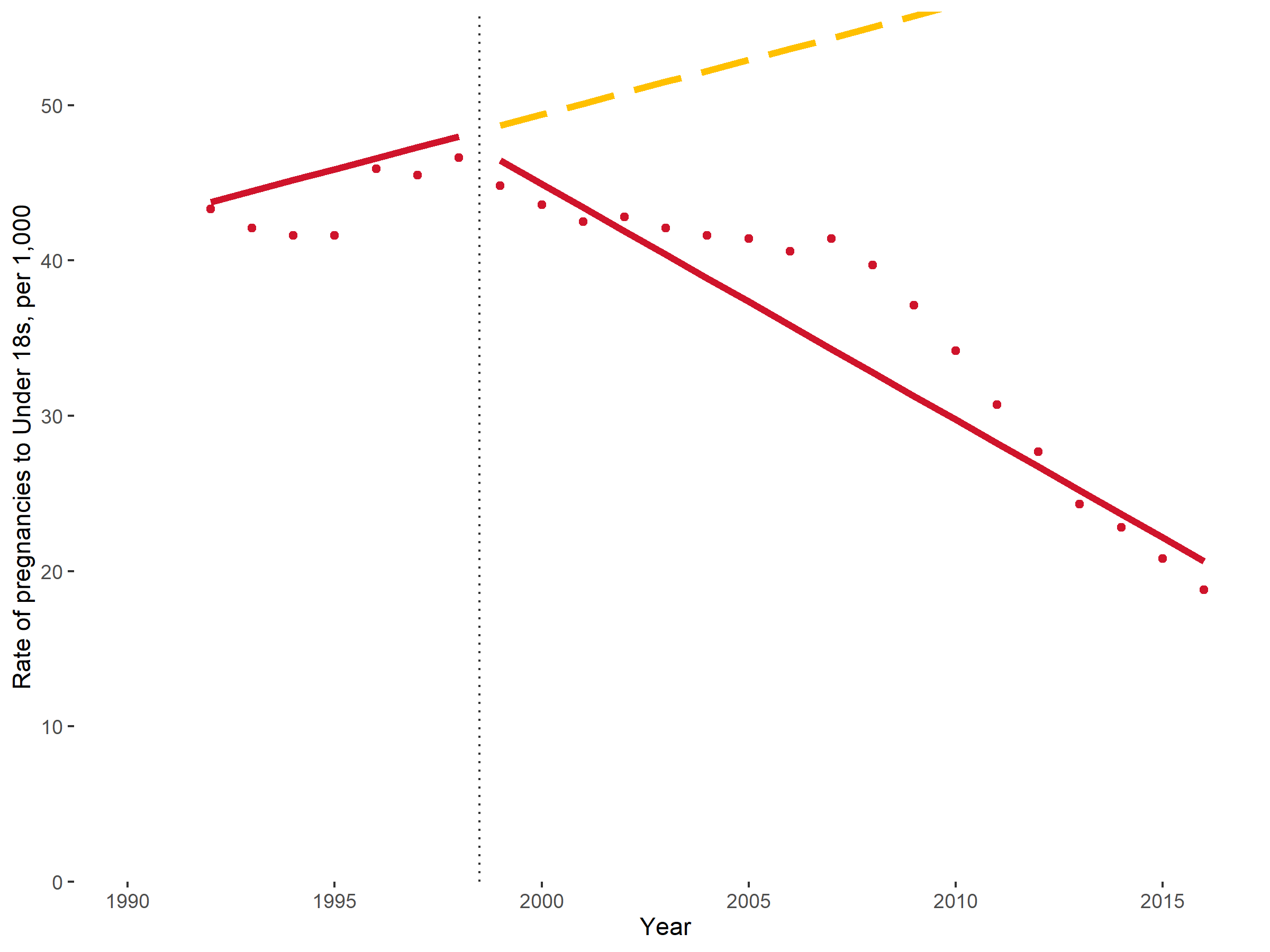


Autocorrelation correction: AR1, MA0

MSPE = 10.6

R^2^ = 0.932

| Coefficient | Value | Std.Error | Lower CI | Upper CI |
| --- | --- | --- | --- | --- |
| England (est) rate at 1991 | 43.060 | 3.463 | 36.273 | 49.847 |
| England base trend | 0.704 | 0.530 | -0.335 | 1.743 |
| England change in level at intervention | -0.032 | 1.553 | -3.076 | 3.012 |
| England change in trend at intervention | -2.221 | 0.646 | -3.488 | -0.954 |

## England 1992 – 2016 with 1996-1998 ‘pill scare’ corrector


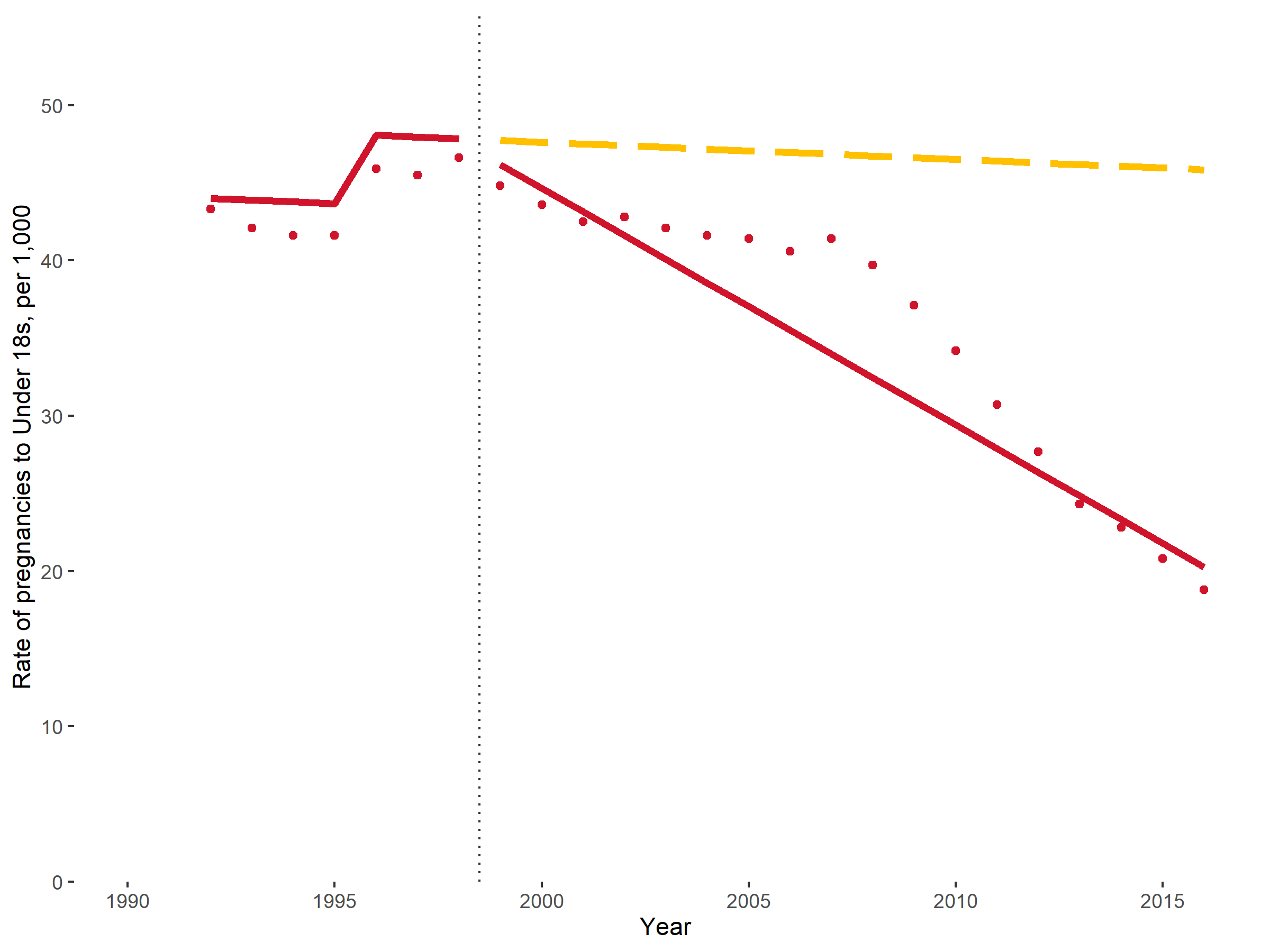


Autocorrelation correction: AR1, MA0

MSPE = 10.8

R^2^ = 0.935

| Coefficient | Value | Std.Error | Lower CI | Upper CI |
| --- | --- | --- | --- | --- |
| England (est) rate at 1991 | 44.110 | 3.335 | 37.573 | 50.647 |
| England base trend | -0.111 | 0.506 | -1.103 | 0.881 |
| England change in level at intervention | -0.144 | 1.265 | -2.623 | 2.336 |
| England change in trend at intervention | -1.412 | 0.596 | -2.580 | -0.244 |
| ‘Pill Scare’ corrector | 4.507 | 1.361 | 1.840 | 7.174 |

## England compared with Scotland 1992 - 2016


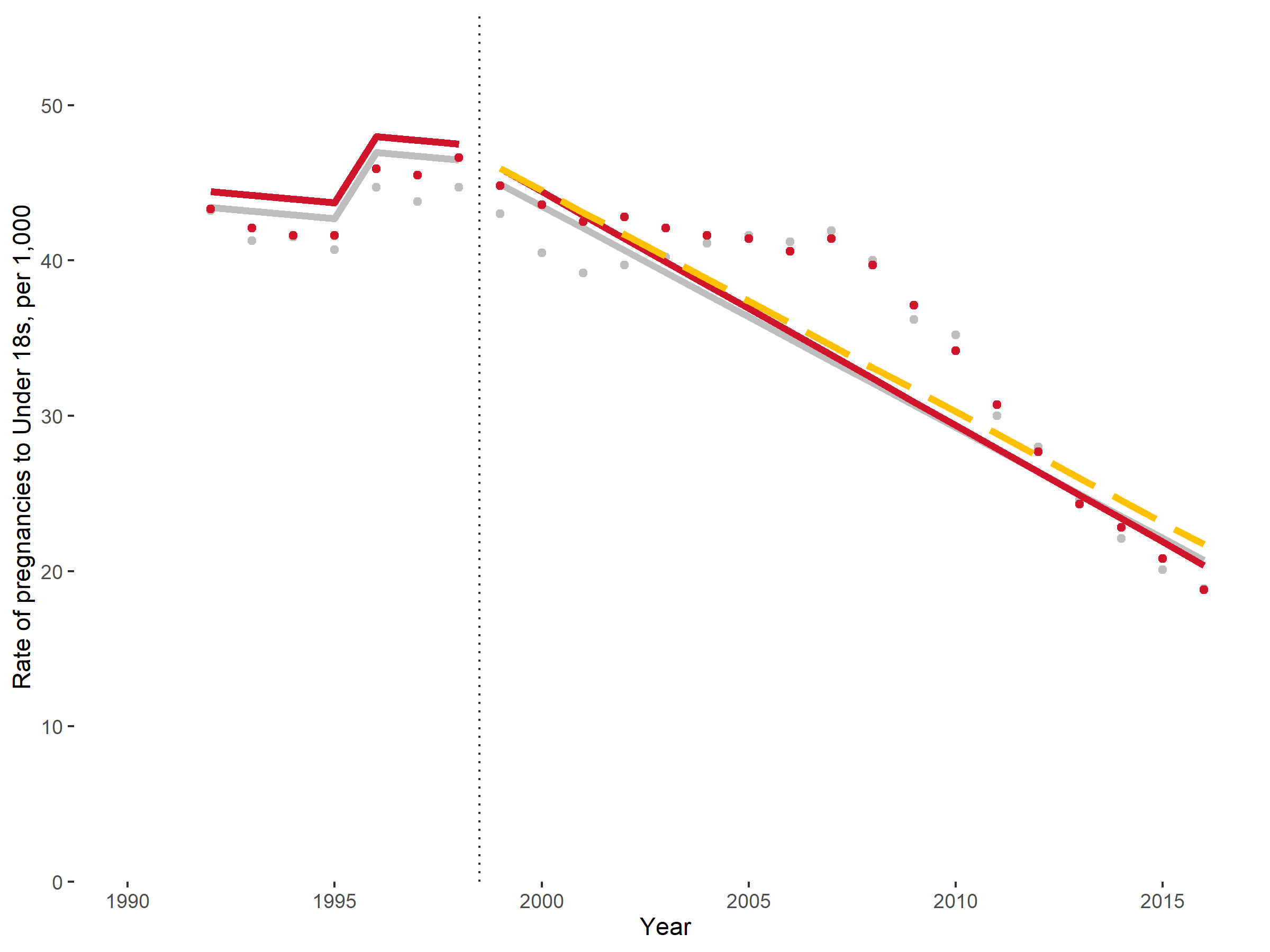


Autocorrelation correction: AR1, MA0

MSPE = 12.4

R^2^ = 0.918

| Coefficient | Value | Std.Error | Lower CI | Upper CI |
| --- | --- | --- | --- | --- |
| Scotland (est) rate at 1991 | 43.651 | 3.285 | 37.213 | 50.089 |
| Scotland base trend | -0.239 | 0.413 | -1.048 | 0.571 |
| England difference in rate at 1991 | 1.018 | 4.121 | -7.059 | 9.094 |
| Scotland change in level at intervention | -0.143 | 1.479 | -3.041 | 2.755 |
| Scotland change in trend at intervention | -1.187 | 0.515 | -2.195 | -0.178 |
| England difference in level from control at intervention | 0.064 | 2.090 | -4.032 | 4.160 |
| England difference in trend from control at intervention | -0.077 | 0.339 | -0.742 | 0.587 |
| ‘Pill Scare’ corrector | 4.509 | 1.129 | 2.295 | 6.723 |

## England compared with Wales 1992 - 2016


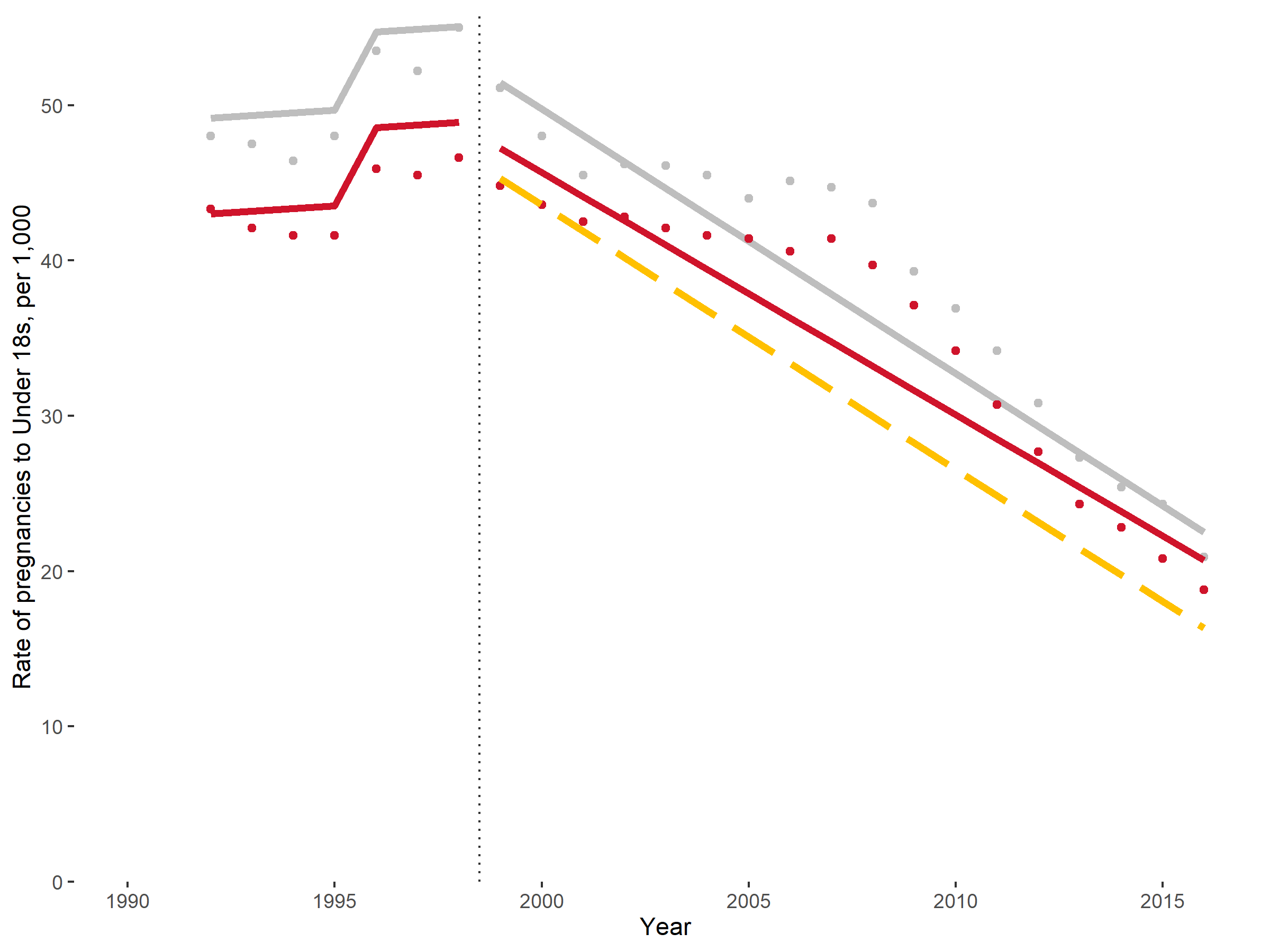


Autocorrelation correction: AR1, MA0

MSPE = 9.42

R^2^ = 0.951

| Coefficient | Value | Std.Error | Lower CI | Upper CI |
| --- | --- | --- | --- | --- |
| Wales (est) rate at 1991 | 48.988 | 2.950 | 43.206 | 54.769 |
| Wales base trend | 0.170 | 0.406 | -0.626 | 0.966 |
| England difference in rate at 1991 | -6.158 | 3.598 | -13.210 | 0.893 |
| Wales change in level at intervention | -1.903 | 1.482 | -4.808 | 1.001 |
| Wales change in trend at intervention | -1.872 | 0.502 | -2.855 | -0.889 |
| England difference in level from control at intervention | 1.807 | 2.093 | -2.295 | 5.908 |
| England difference in trend from control at intervention | 0.143 | 0.315 | -0.475 | 0.760 |
| ‘Pill Scare’ corrector | 4.864 | 1.139 | 2.631 | 7.097 |

## England compared with Scotland 1992 – 2016 with 2008 common shock


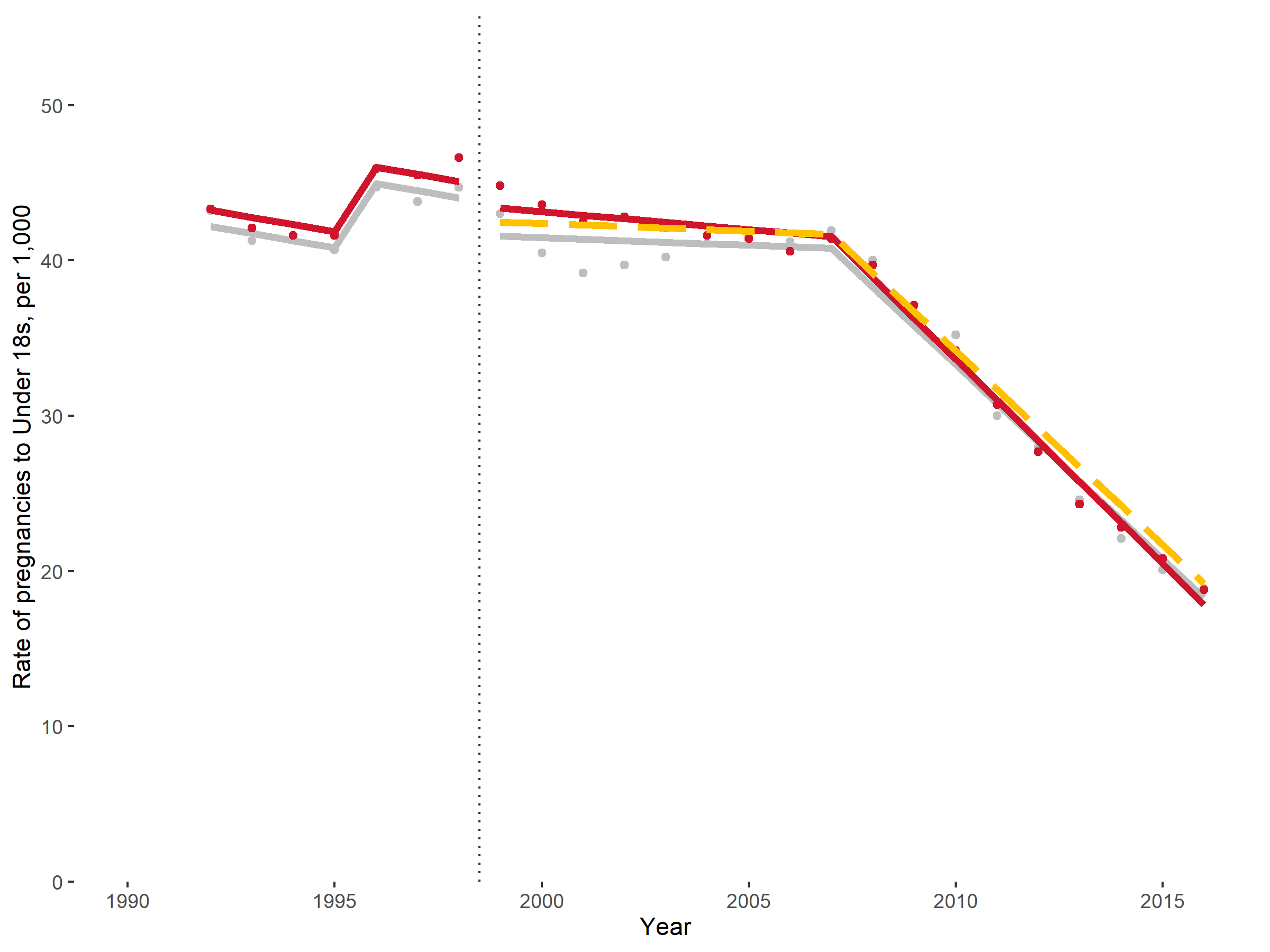


Autocorrelation correction: AR1, MA0

MSPE = 0.793

R^2^ = 0.994

| Coefficient | Value | Std.Error | Lower CI | Upper CI |
| --- | --- | --- | --- | --- |
| Scotland (est) rate at 1991 | 42.654 | 0.872 | 40.945 | 44.363 |
| Scotland base trend | -0.460 | 0.225 | -0.902 | -0.019 |
| England difference in rate at 1991 | 1.040 | 0.757 | -0.443 | 2.523 |
| Scotland change in level at intervention | -2.376 | 0.897 | -4.134 | -0.619 |
| Scotland change in trend at intervention | 0.363 | 0.265 | -0.157 | 0.883 |
| England difference in level from control at intervention | 0.896 | 1.104 | -1.267 | 3.059 |
| England difference in trend from control at intervention | -0.131 | 0.088 | -0.304 | 0.041 |
| Change in trend at 2008 common shock | -2.403 | 0.172 | -2.740 | -2.065 |
| ‘Pill Scare’ corrector | 4.610 | 0.855 | 2.933 | 6.287 |

## England compared with Wales 1992 - 2016 with 2008 common shock


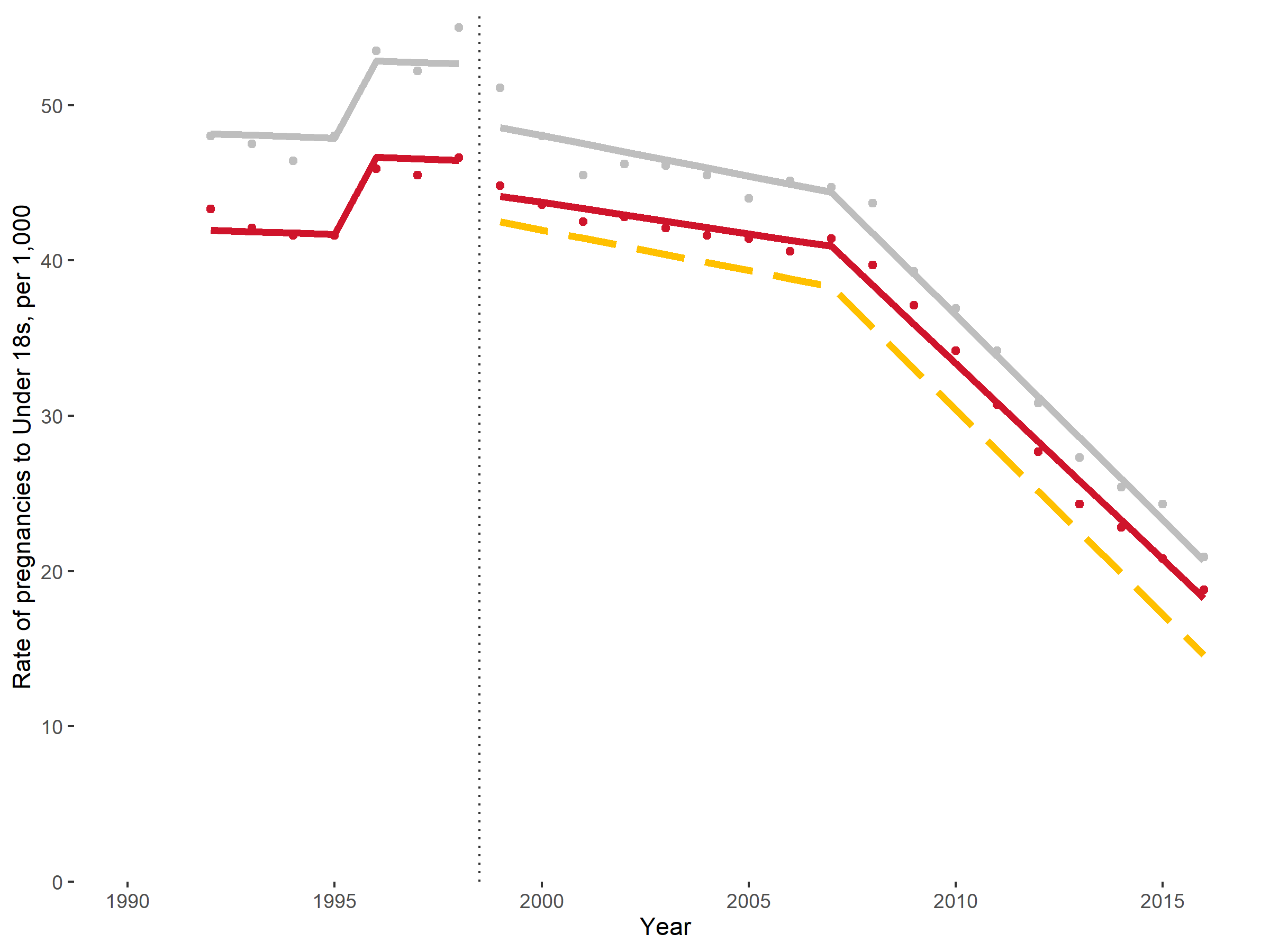


Autocorrelation correction: AR1, MA0

MSPE = 0.863

R^2^ = 0.995

| Coefficient | Value | Std.Error | Lower CI | Upper CI |
| --- | --- | --- | --- | --- |
| Wales (est) rate at 1991 | 48.244 | 0.894 | 46.492 | 49.995 |
| Wales base trend | -0.091 | 0.246 | -0.573 | 0.391 |
| England difference in rate at 1991 | -6.200 | 0.741 | -7.653 | -4.748 |
| Wales change in level at intervention | -3.575 | 0.944 | -5.425 | -1.726 |
| Wales change in trend at intervention | -0.430 | 0.280 | -0.980 | 0.119 |
| England difference in level from control at intervention | 1.666 | 1.127 | -0.543 | 3.874 |
| England difference in trend from control at intervention | 0.117 | 0.087 | -0.054 | 0.288 |
| Change in trend at 2008 common shock | -2.113 | 0.172 | -2.449 | -1.777 |
| ‘Pill Scare’ corrector | 5.050 | 0.957 | 3.175 | 6.925 |

## England and Wales compared with Scotland 1987 - 2016


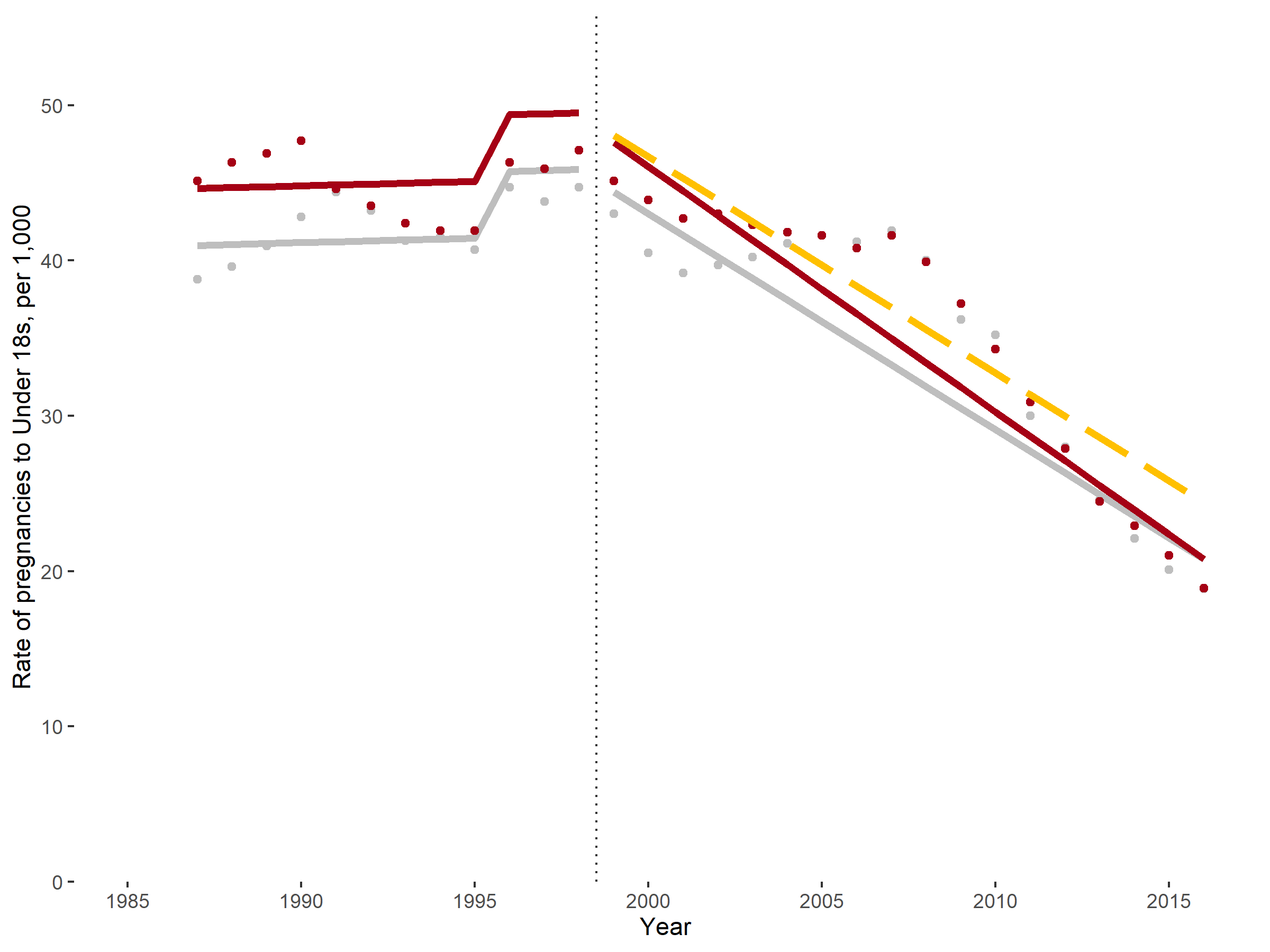


Autocorrelation correction: AR1, MA0

MSPE = 10.5

R^2^ = 0.924

| Coefficient | Value | Std.Error | Lower CI | Upper CI |
| --- | --- | --- | --- | --- |
| Scotland (est) rate at 1986 | 40.918 | 2.970 | 35.098 | 46.739 |
| Scotland base trend | 0.058 | 0.261 | -0.454 | 0.570 |
| England and Wales difference in rate at 1986 | 3.660 | 3.518 | -3.235 | 10.554 |
| Scotland change in level at intervention | -0.061 | 1.506 | -3.013 | 2.891 |
| Scotland change in trend at intervention | -1.449 | 0.389 | -2.211 | -0.686 |
| England and Wales difference in level from control at intervention | -0.258 | 2.127 | -4.426 | 3.910 |
| England and Wales difference in trend from control at intervention | -0.188 | 0.322 | -0.818 | 0.443 |
| ‘Pill Scare’ corrector | 4.238 | 1.100 | 2.081 | 6.395 |

## England and Wales compared with Scotland 1987 - 2016 with 2008 common shock


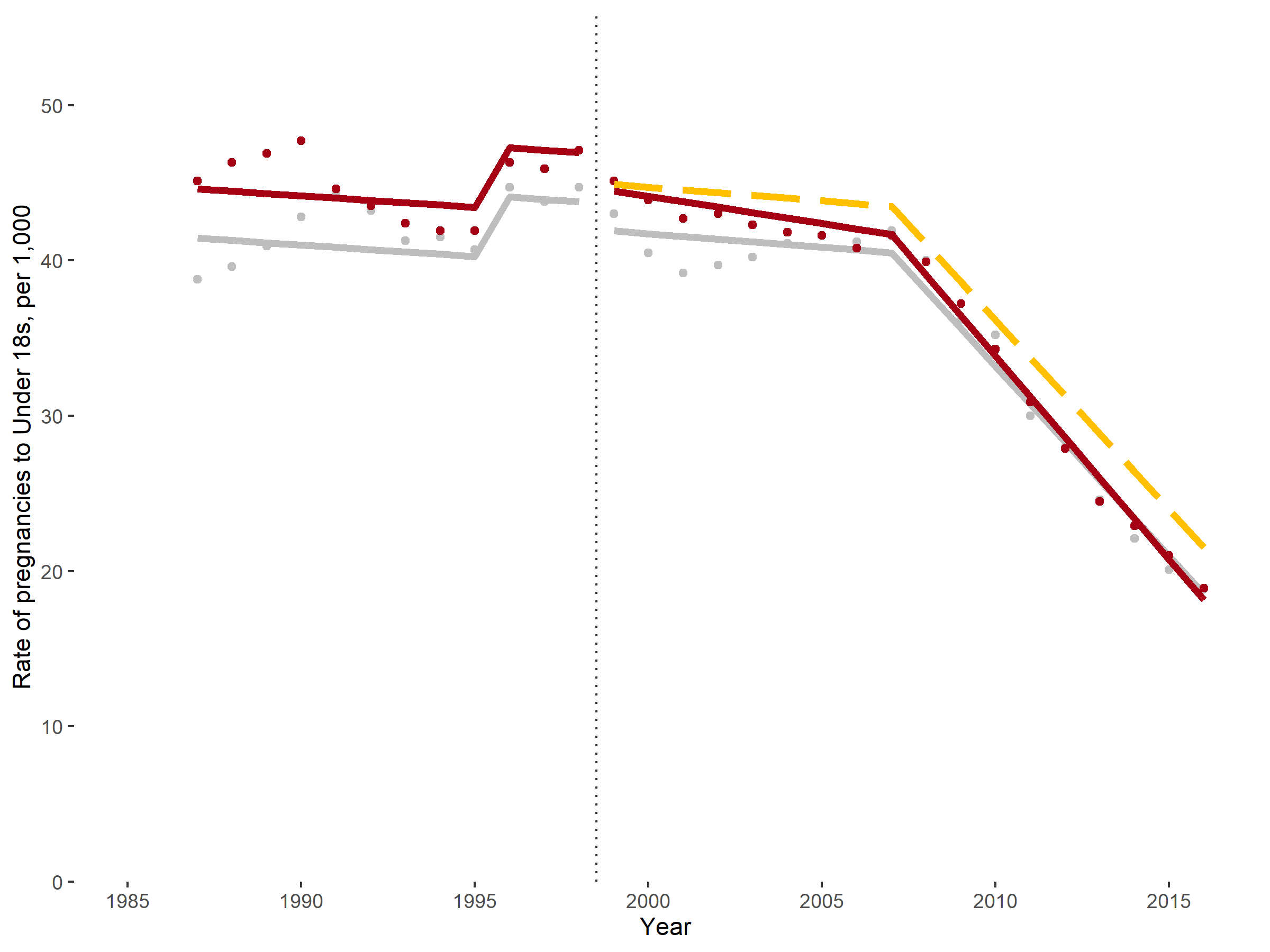


Autocorrelation correction: AR1, MA0

MSPE = 1.77

R^2^ = 0.986

| Coefficient | Value | Std.Error | Lower CI | Upper CI |
| --- | --- | --- | --- | --- |
| Scotland (est) rate at 1986 | 41.591 | 1.181 | 39.277 | 43.906 |
| Scotland base trend | -0.148 | 0.149 | -0.439 | 0.143 |
| England and Wales difference in rate at 1986 | 3.167 | 1.132 | 0.948 | 5.386 |
| Scotland change in level at intervention | -1.709 | 1.143 | -3.950 | 0.533 |
| Scotland change in trend at intervention | -0.027 | 0.257 | -0.530 | 0.476 |
| England and Wales difference in level from control at intervention | -0.414 | 1.506 | -3.365 | 2.538 |
| England and Wales difference in trend from control at intervention | -0.175 | 0.144 | -0.457 | 0.108 |
| Change in trend at 2008 common shock | -2.266 | 0.273 | -2.802 | -1.730 |
| ‘Pill Scare’ corrector | 3.975 | 0.870 | 2.269 | 5.680 |

## England and Wales under-20 pregnancies compared with Scotland 1990 - 2016


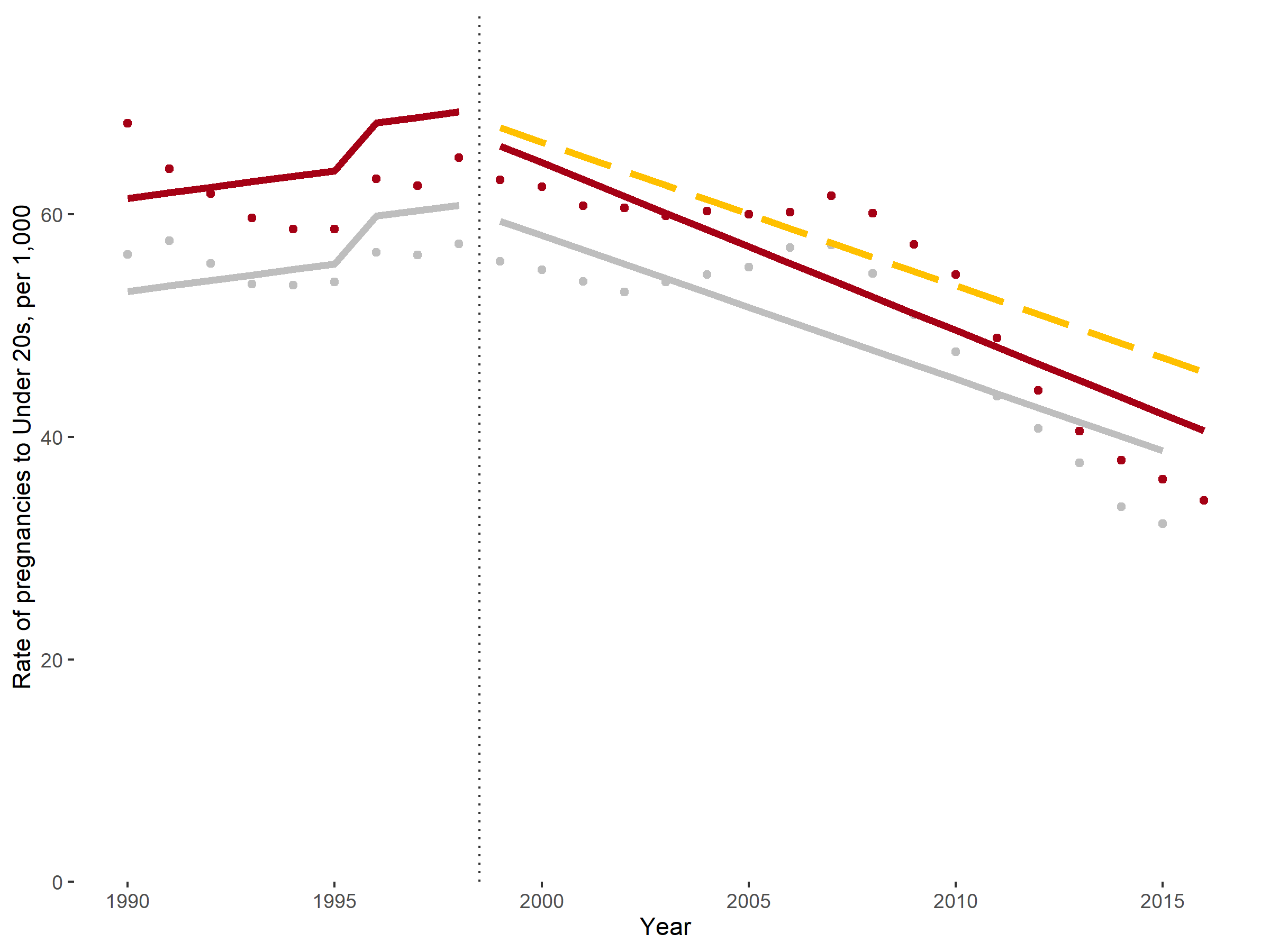


Autocorrelation correction: AR2, MA1

MSPE = 18.2

R^2^ = 0.875

| Coefficient | Value | Std.Error | Lower CI | Upper CI |
| --- | --- | --- | --- | --- |
| Scotland (est) rate at 1989 | 52.597 | 2.001 | 48.675 | 56.519 |
| Scotland base trend | 0.493 | 0.318 | -0.131 | 1.116 |
| England and Wales difference in rate at 1989 | 8.382 | 0.968 | 6.486 | 10.278 |
| Scotland change in level at intervention | -0.145 | 1.223 | -2.541 | 2.252 |
| Scotland change in trend at intervention | -1.784 | 0.420 | -2.607 | -0.960 |
| England and Wales difference in level from control at intervention | -1.414 | 1.717 | -4.780 | 1.951 |
| England and Wales difference in trend from control at intervention | -0.216 | 0.163 | -0.535 | 0.104 |
| ‘Pill Scare’ corrector | 3.822 | 0.873 | 2.110 | 5.533 |

## England and Wales under-20 pregnancies compared with Scotland 1990 - 2016 with 2008 common shock


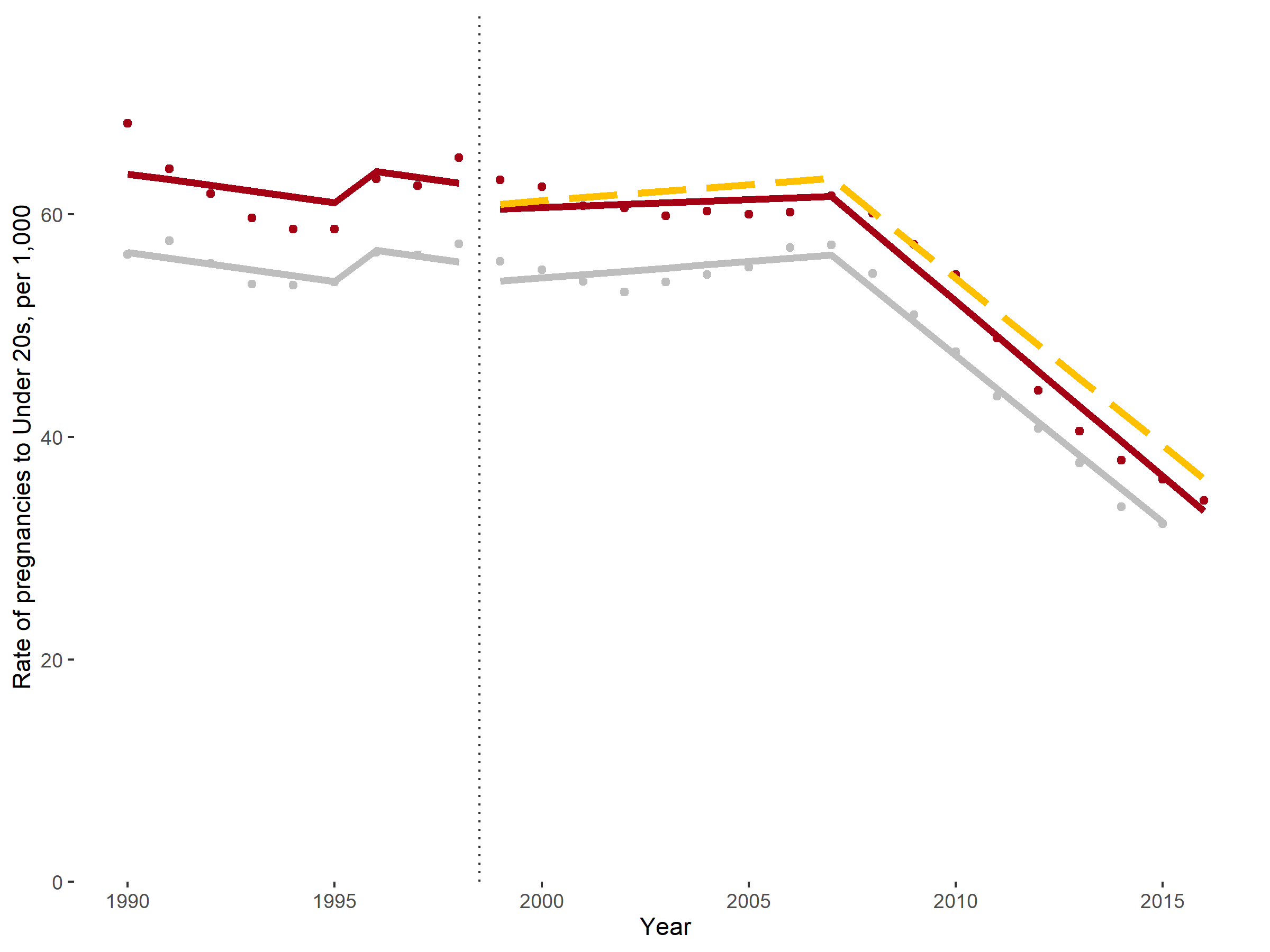


Autocorrelation correction: AR2, MA1

MSPE = 2.14

R^2^ = 0.986

| Coefficient | Value | Std.Error | Lower CI | Upper CI |
| --- | --- | --- | --- | --- |
| Scotland (est) rate at 1989 | 57.123 | 0.683 | 55.784 | 58.462 |
| Scotland base trend | -0.521 | 0.155 | -0.825 | -0.217 |
| England and Wales difference in rate at 1989 | 7.056 | 0.268 | 6.530 | 7.582 |
| Scotland change in level at intervention | -2.015 | 0.757 | -3.498 | -0.532 |
| Scotland change in trend at intervention | 0.810 | 0.169 | 0.479 | 1.142 |
| England and Wales difference in level from control at intervention | -0.441 | 0.617 | -1.651 | 0.768 |
| England and Wales difference in trend from control at intervention | -0.146 | 0.046 | -0.237 | -0.055 |
| Change in trend at 2008 common shock | -3.291 | 0.141 | -3.568 | -3.015 |
| ‘Pill Scare’ corrector | 3.326 | 0.783 | 1.792 | 4.859 |

## England and Wales under-16 pregnancies compared with Scotland 1994 - 2016


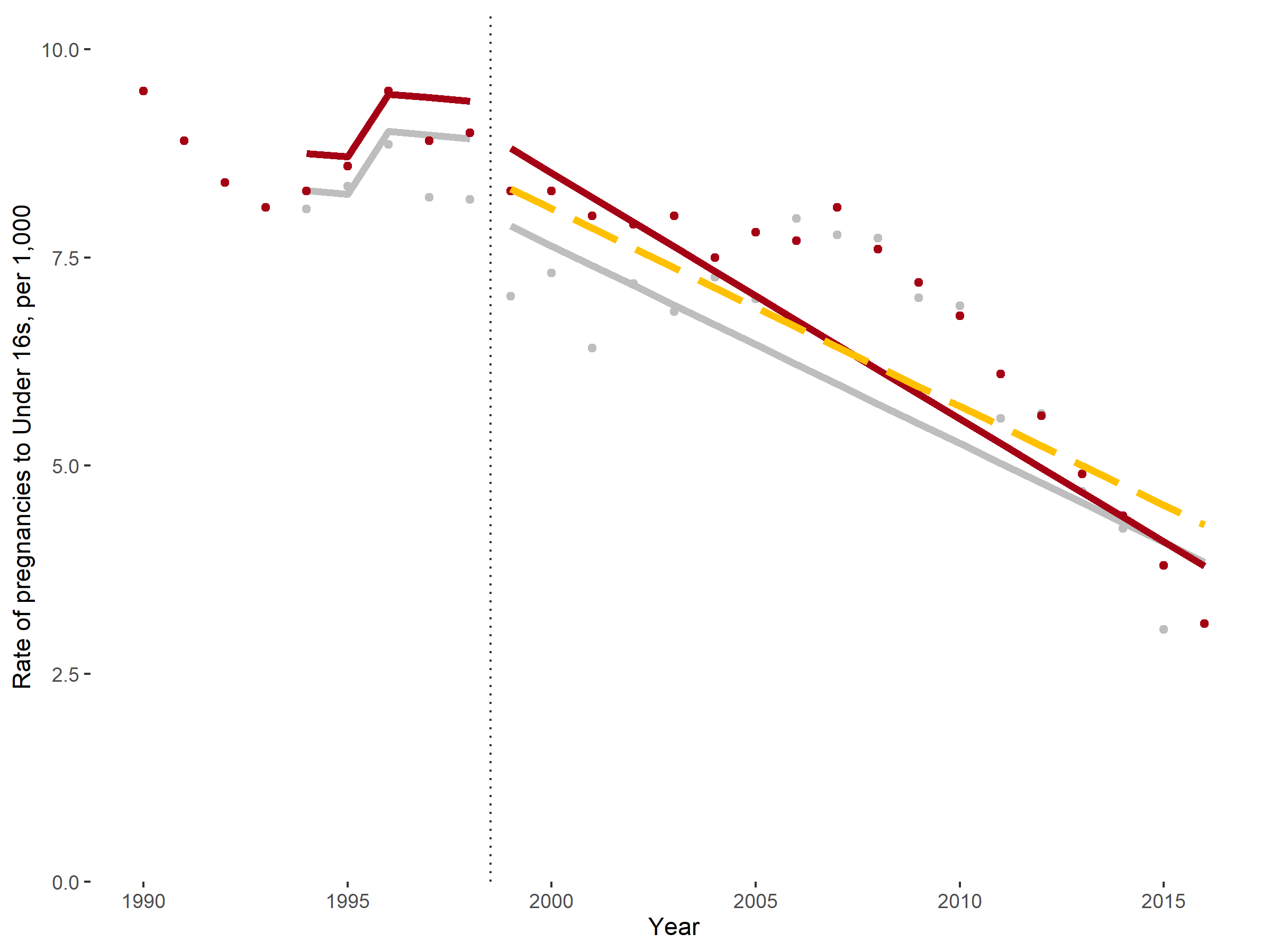


Autocorrelation correction: AR1, MA1

MSPE = 0.74

R^2^ = 0.882

| Coefficient | Value | Std.Error | Lower CI | Upper CI |
| --- | --- | --- | --- | --- |
| Scotland (est) rate at 1993 | 8.348 | 0.898 | 6.587 | 10.108 |
| Scotland base trend | -0.043 | 0.192 | -0.419 | 0.333 |
| England and Wales difference in rate at 1993 | 0.447 | 1.061 | -1.633 | 2.527 |
| Scotland change in level at intervention | -0.814 | 0.525 | -1.842 | 0.214 |
| Scotland change in trend at intervention | -0.195 | 0.215 | -0.615 | 0.226 |
| England and Wales difference in level from control at intervention | 0.543 | 0.738 | -0.905 | 1.990 |
| England and Wales difference in trend from control at intervention | -0.058 | 0.097 | -0.247 | 0.132 |
| ‘Pill Scare’ corrector | 0.796 | 0.442 | -0.070 | 1.661 |

## England and Wales under-16 pregnancies compared with Scotland 1994 - 2016 with 2008 common shock


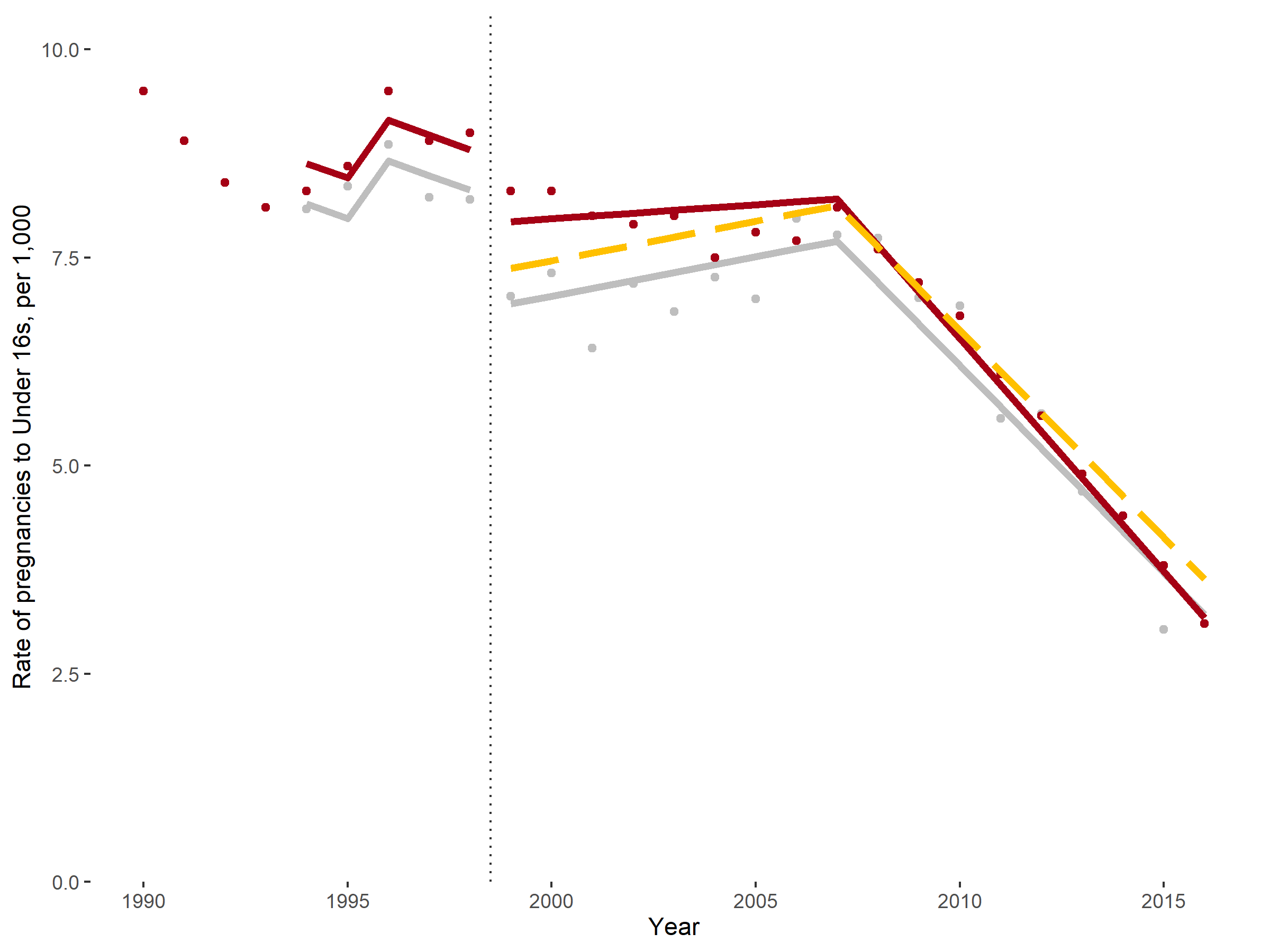


Autocorrelation correction: AR1, MA1

MSPE = 0.0983

R^2^ = 0.982

| Coefficient | Value | Std.Error | Lower CI | Upper CI |
| --- | --- | --- | --- | --- |
| Scotland (est) rate at 1993 | 8.321 | 0.332 | 7.670 | 8.972 |
| Scotland base trend | -0.176 | 0.140 | -0.450 | 0.097 |
| England and Wales difference in rate at 1993 | 0.486 | 0.279 | -0.060 | 1.033 |
| Scotland change in level at intervention | -1.461 | 0.330 | -2.108 | -0.814 |
| Scotland change in trend at intervention | 0.271 | 0.147 | -0.017 | 0.559 |
| England and Wales difference in level from control at intervention | 0.564 | 0.387 | -0.195 | 1.324 |
| England and Wales difference in trend from control at intervention | -0.061 | 0.029 | -0.117 | -0.004 |
| Change in trend at 2008 common shock | -0.593 | 0.055 | -0.701 | -0.485 |
| ‘Pill Scare’ corrector | 0.870 | 0.392 | 0.101 | 1.639 |

# Synthetic Control analyses outputs

Outputted from [[link anonymised]](https://github.com/andrewbaxter439/teen-preg-project/blob/master/Synth-report-updated.Rmd)

## Setting up data

Data from Human Mortality Database and Human Fertility Database was used to calculate birth rates. This was tidied to give Under-18 rates:

| Code | Country | Year | rate | GDPperCap | MF_ratio | MobilePhones | UrbanPop |
| --- | --- | --- | --- | --- | --- | --- | --- |
| 5 | Denmark | 1990 | 2.94 | 26891.44 | 1.04 | 2.88 | 84.84 |
| 5 | Denmark | 1991 | 2.65 | 27011.39 | 1.04 | 3.41 | 84.87 |
| 5 | Denmark | 1992 | 3.08 | 29569.65 | 1.04 | 4.08 | 84.90 |
| 5 | Denmark | 1993 | 2.90 | 27597.97 | 1.04 | 6.89 | 84.92 |
| 5 | Denmark | 1994 | 2.71 | 29995.57 | 1.05 | 9.66 | 84.95 |
| 5 | Denmark | 1995 | 2.19 | 35351.38 | 1.05 | 15.71 | 84.98 |

Abortion estimates were added to give Under-20 rates:

| Code | Country | Year | pRate | rate | GDPperCap | MF_ratio | MobilePhones | UrbanPop |
| --- | --- | --- | --- | --- | --- | --- | --- | --- |
| 4 | Denmark | 1990 | 26.51 | 9.20 | 26891.44 | 1.05 | 2.88 | 84.84 |
| 4 | Denmark | 1991 | 25.33 | 9.00 | 27011.39 | 1.04 | 3.41 | 84.87 |
| 4 | Denmark | 1992 | 25.43 | 9.70 | 29569.65 | 1.04 | 4.08 | 84.90 |
| 4 | Denmark | 1993 | 24.81 | 9.14 | 27597.97 | 1.05 | 6.89 | 84.92 |
| 4 | Denmark | 1994 | 24.07 | 9.21 | 29995.57 | 1.05 | 9.66 | 84.95 |
| 4 | Denmark | 1995 | 23.08 | 8.72 | 35351.38 | 1.05 | 15.71 | 84.98 |

## Iterating through year combinations

For each comparison, I iterated through all combinations of years as special

Predictors to minimise MSPE (whilst prioritising fewest groupings). For example, for the under-18 basic model with years as special

Predictors:

it_u18_sp <- testSynthIterations(
 yrs = 1990:1998,
 pred = "rate",
 data = synthData_u18[,1:4],
 ccodes = u_18_ccodes,
 n = 4,

Predictors = NULL,
 time.optimise = 1985:1998
) %>%
 arrange(groups, mspe)

## All countries, no additional Predictors

### Model 1: Under-18 birth rates

#### All Countries graph


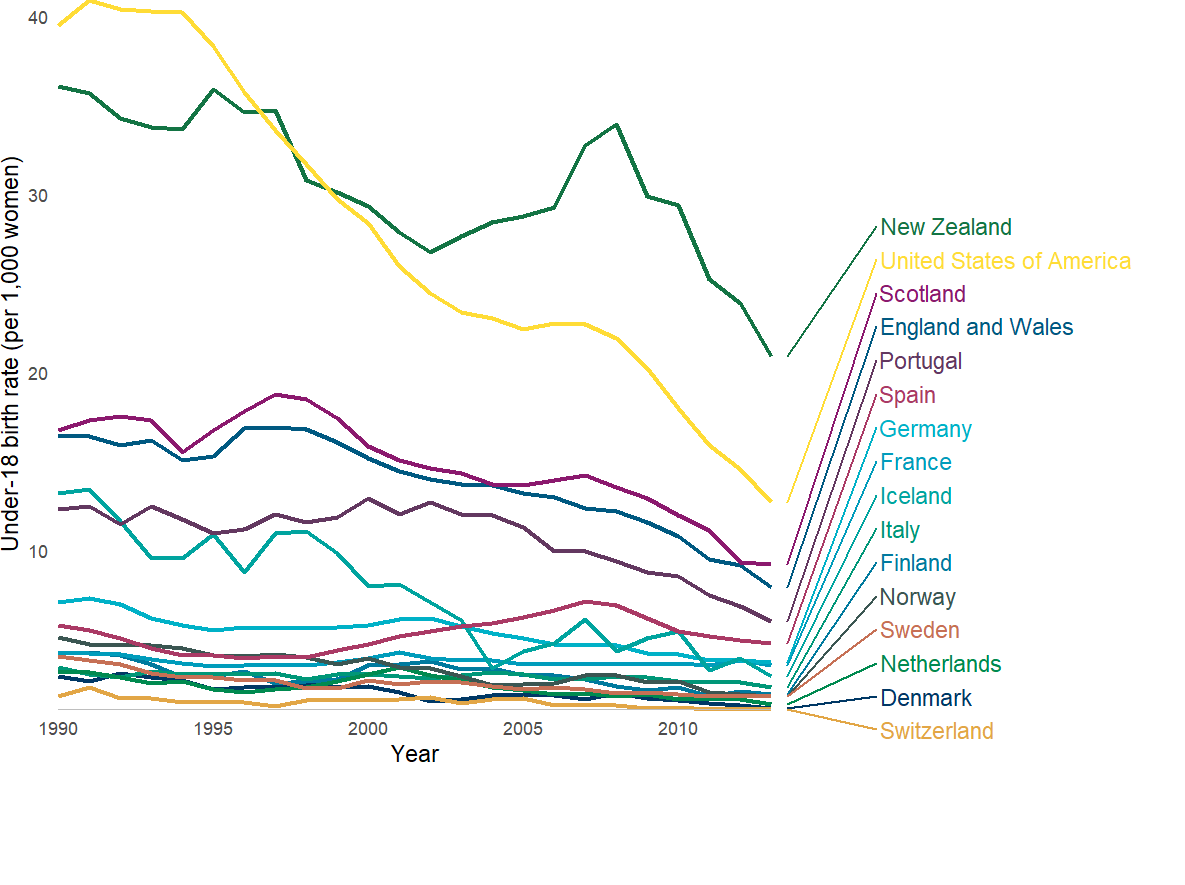


#### England vs Synthetic Control

Over the period 1999 to 2013, England and Wales saw a difference of -3,744 births (to an average population of 995,340 women).


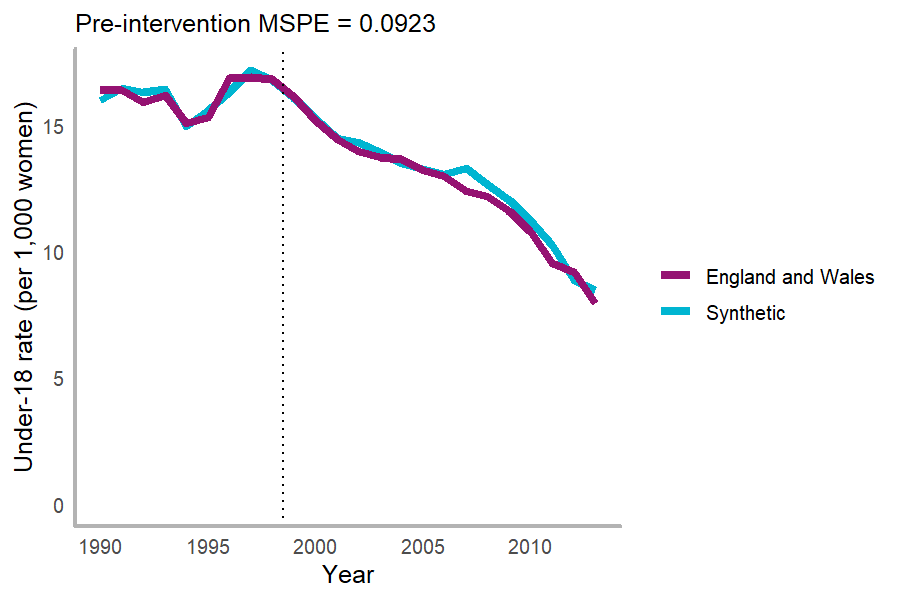


#### Weights and balance

Country weights

| Country | Weight |
| --- | --- |
| Scotland | 0.672 |
| Portugal | 0.295 |
| United States of America | 0.016 |
| New Zealand | 0.012 |
| Iceland | 0.001 |
| Sweden | 0.001 |
| Switzerland | 0.000 |
| Germany | 0.000 |
| Denmark | 0.000 |
| Spain | 0.000 |
| Finland | 0.000 |
| France | 0.000 |
| Italy | 0.000 |
| Netherlands | 0.000 |
| Norway | 0.000 |

Predictor weights

|  | v.weights |
| --- | --- |
| special.rate.1990.1993 | 0.01 |
| special.rate.1994 | 0.333 |
| special.rate.1995 | 0.295 |
| special.rate.1996.1998 | 0.362 |

Predictor balance between synthetic and treated units

|  | Treated | Synthetic | Sample Mean |
| --- | --- | --- | --- |
| special.rate.1990.1993 | 16.214 | 16.275 | 10.415 |
| special.rate.1994 | 15.078 | 14.952 | 9.629 |
| special.rate.1995 | 15.285 | 15.561 | 9.660 |
| special.rate.1996.1998 | 16.836 | 16.728 | 9.257 |

#### Placebo testing by country and time


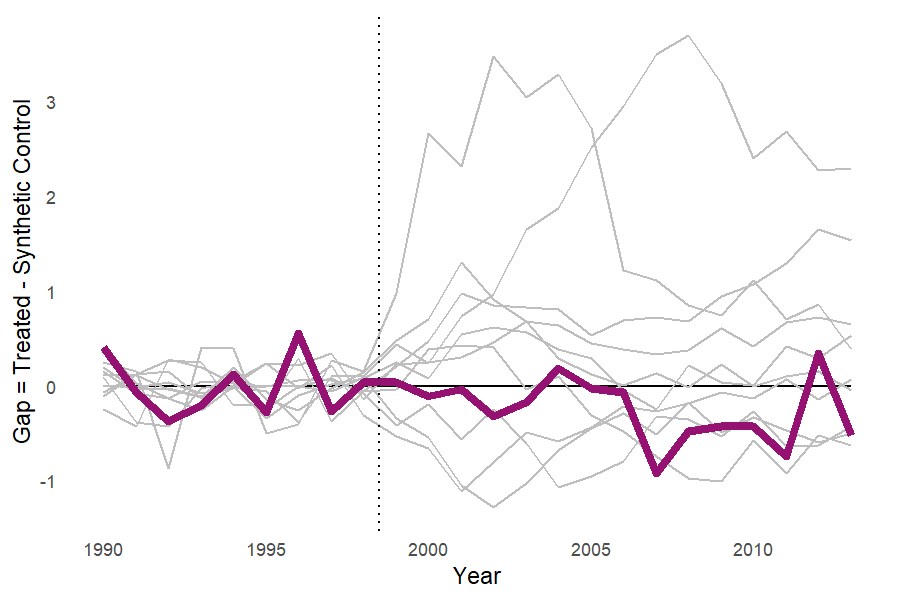


In placebo-country tests, England and Wales ranked 14 out of 16 countries by pre/post-MSPE ratio.


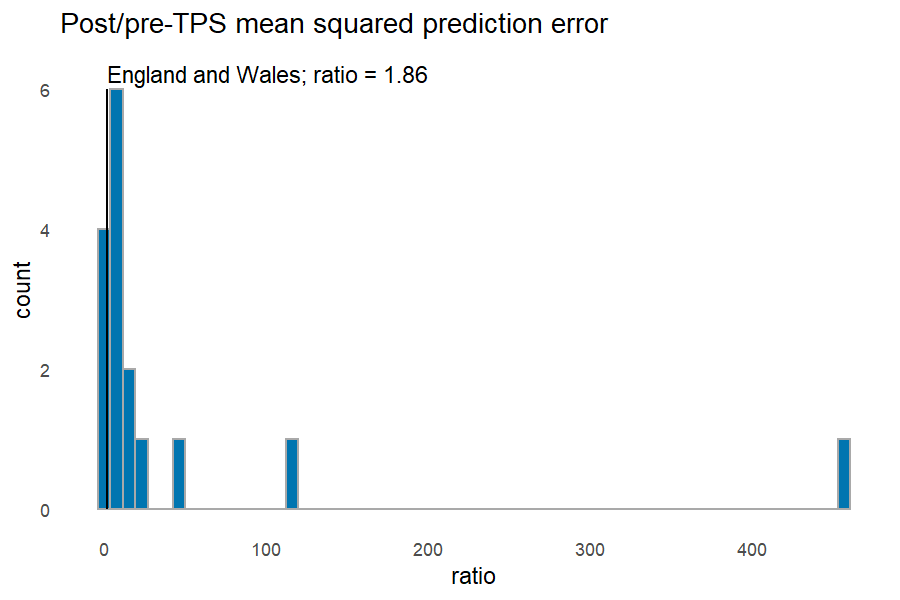


### Model 2: Under-20 pregnancy rates

#### All Countries graph


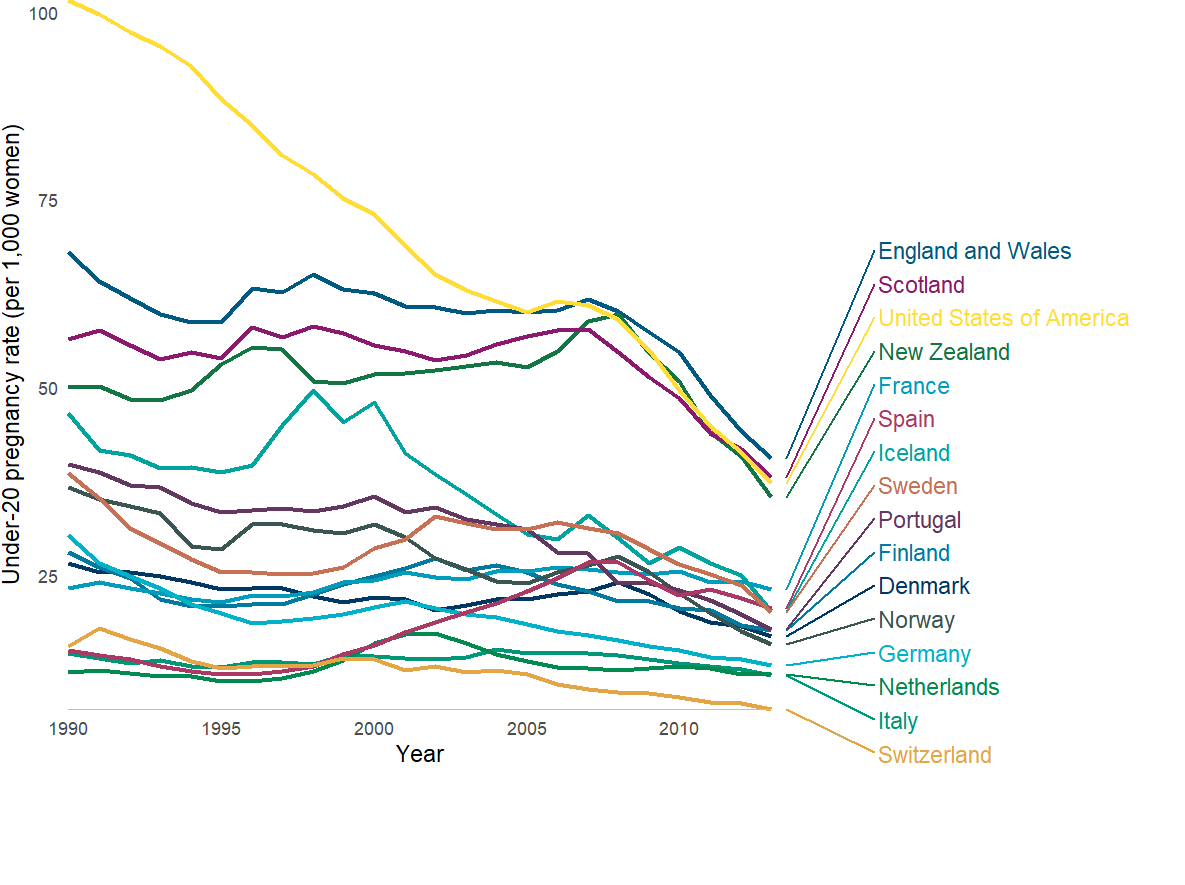


#### England vs Synthetic Control

Over the period 1999 to 2013, England and Wales saw a difference of 152,471 pregnancies (to an average population of 1,668,190 women).


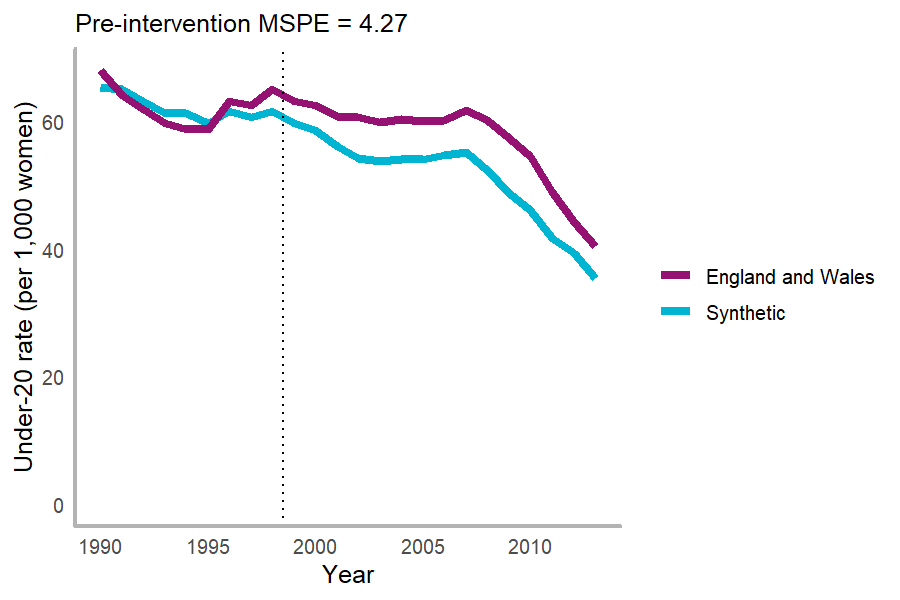


#### Weights and balance

Country weights

| Country | Weight |
| --- | --- |
| Scotland | 0.639 |
| United States of America | 0.227 |
| Iceland | 0.134 |
| Switzerland | 0.000 |
| Germany | 0.000 |
| Denmark | 0.000 |
| Spain | 0.000 |
| Finland | 0.000 |
| France | 0.000 |
| Italy | 0.000 |
| Netherlands | 0.000 |
| Norway | 0.000 |
| Portugal | 0.000 |
| New Zealand | 0.000 |
| Sweden | 0.000 |

Predictor weights

|  | v.weights |
| --- | --- |
| special.pRate.1990 | 0.152 |
| special.pRate.1991.1995 | 0.578 |
| special.pRate.1996.1998 | 0.271 |

Predictor balance between synthetic and treated units

|  | Treated | Synthetic | Sample Mean |
| --- | --- | --- | --- |
| special.pRate.1990 | 68.000 | 65.337 | 35.661 |
| special.pRate.1991.1995 | 60.620 | 62.055 | 32.224 |
| special.pRate.1996.1998 | 63.633 | 61.259 | 30.895 |

#### Placebo testing by country and time


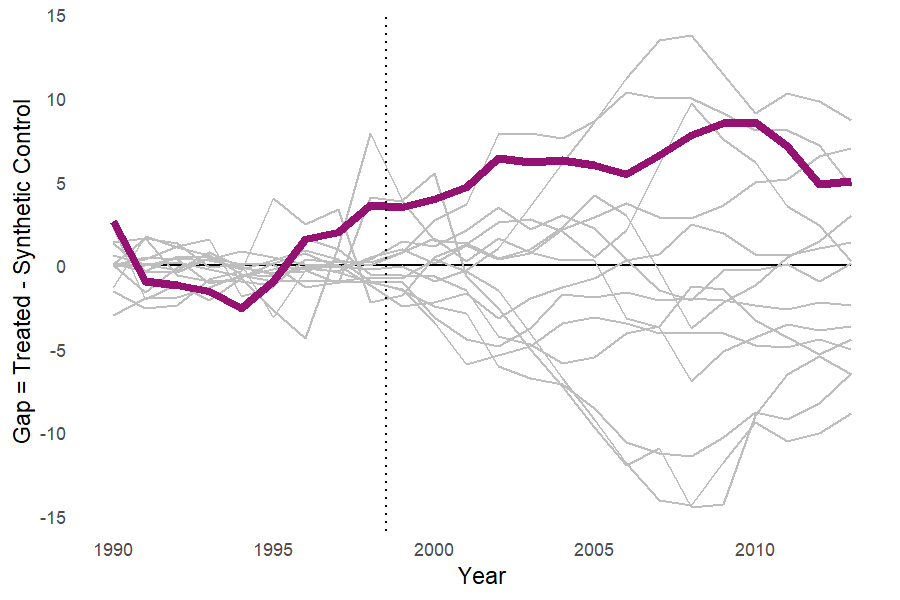


In placebo-country tests, England and Wales ranked 11 out of 16 countries by pre/post-MSPE ratio.


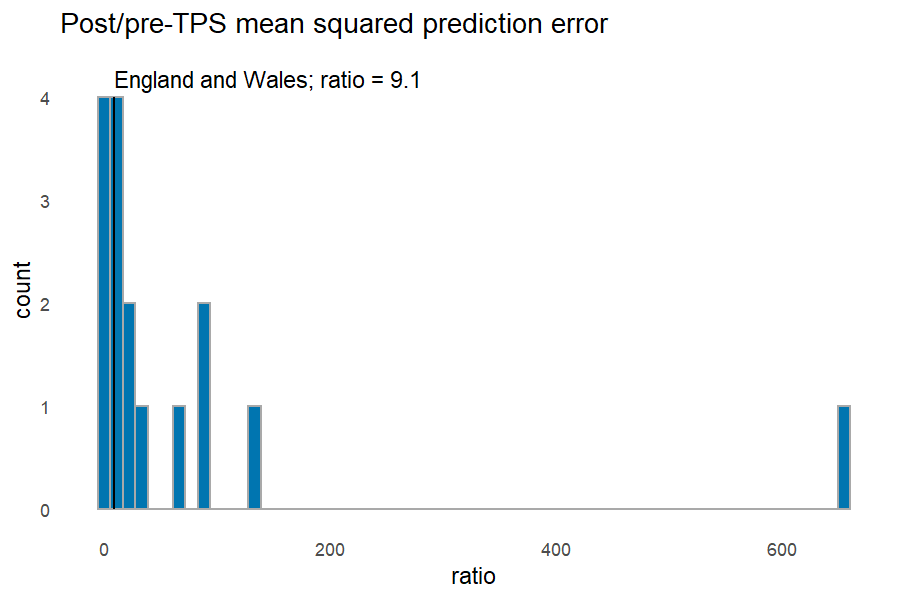


## Filtered to remove Scotland, no additional predictors

### Model 3: Under-18 birth rates

#### England vs Synthetic Control

Over the period 1999 to 2013, England and Wales saw a difference of -17,765 births (to an average population of 995,340 women).


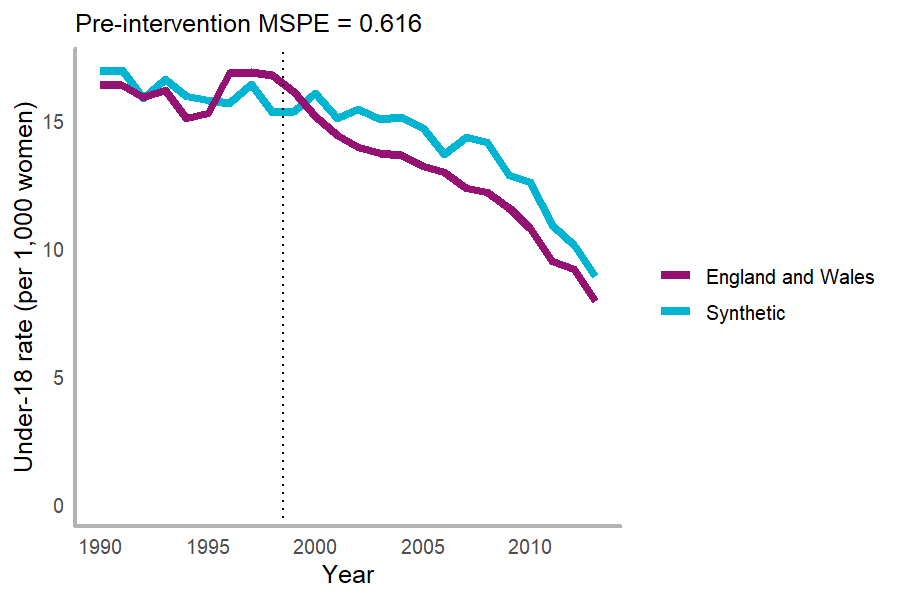


Loss-V (For optimised period) = 0.616, MSPE (for whole pre-intervention observation period) = 0.616.

#### Weights and balance

Country weights

| Country | Weight |
| --- | --- |
| Portugal | 0.807 |
| New Zealand | 0.193 |
| Switzerland | 0.000 |
| Germany | 0.000 |
| Denmark | 0.000 |
| Spain | 0.000 |
| Finland | 0.000 |
| France | 0.000 |
| Iceland | 0.000 |
| Italy | 0.000 |
| Netherlands | 0.000 |
| Norway | 0.000 |
| Sweden | 0.000 |
| United States of America | 0.000 |

Predictor weights

|  | v.weights |
| --- | --- |
| special.rate.1990.1993 | 0.206 |
| special.rate.1994 | 0.156 |
| special.rate.1995 | 0.318 |
| special.rate.1996.1998 | 0.32 |

Predictor balance between synthetic and treated units

|  | Treated | Synthetic | Sample Mean |
| --- | --- | --- | --- |
| special.rate.1990.1993 | 16.214 | 16.587 | 9.928 |
| special.rate.1994 | 15.078 | 15.963 | 9.210 |
| special.rate.1995 | 15.285 | 15.782 | 9.155 |
| special.rate.1996.1998 | 16.836 | 15.803 | 8.607 |

#### Placebo testing by country and time


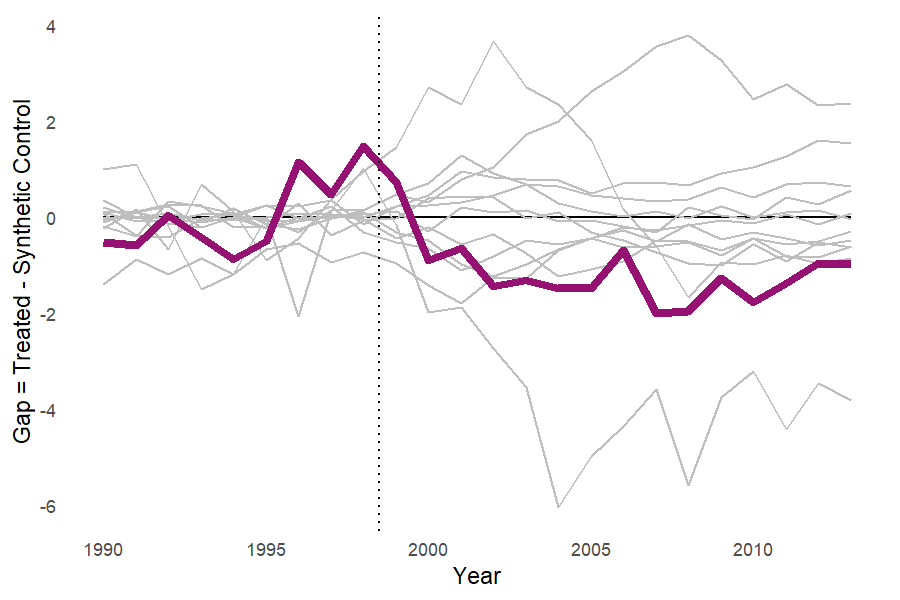


In placebo-country tests, England and Wales ranked 14 out of 16 countries by pre/post-MSPE ratio.


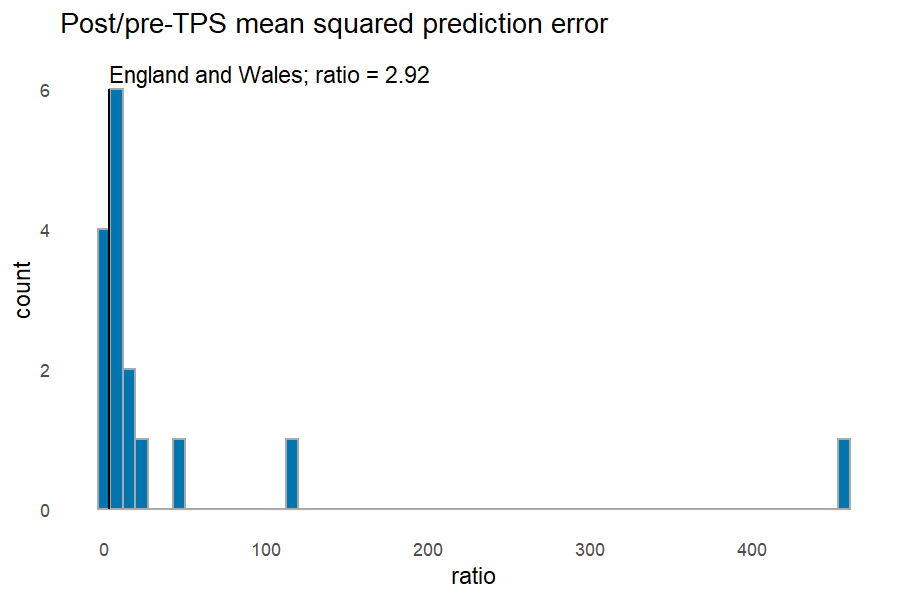


### Model 4: Under-20 pregnancy rates

#### England vs Synthetic Control

Over the period 1999 to 2013, England and Wales saw a difference of 250,707 pregnancies (to an average population of 1,668,190 women).


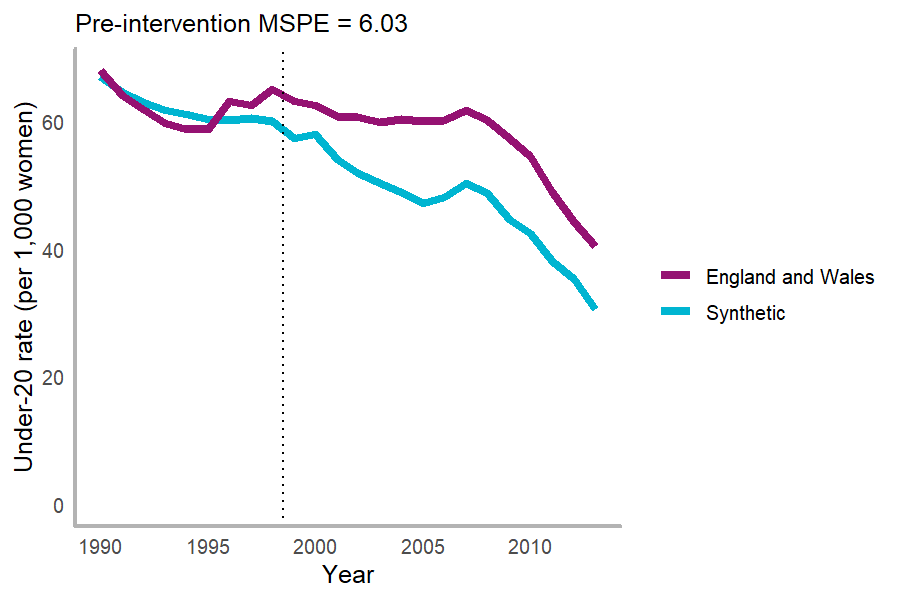


Loss-V (For optimised period) = 6.03, MSPE (for whole pre-intervention observation period) = 6.03.

#### Weights and balance

Country weights

| Country | Weight |
| --- | --- |
| Iceland | 0.359 |
| United States of America | 0.353 |
| New Zealand | 0.288 |
| Switzerland | 0.000 |
| Germany | 0.000 |
| Denmark | 0.000 |
| Spain | 0.000 |
| Finland | 0.000 |
| France | 0.000 |
| Italy | 0.000 |
| Netherlands | 0.000 |
| Norway | 0.000 |
| Portugal | 0.000 |
| Sweden | 0.000 |

Predictor weights

|  | v.weights |
| --- | --- |
| special.pRate.1990 | 0.309 |
| special.pRate.1991.1995 | 0.498 |
| special.pRate.1996.1998 | 0.193 |

Predictor balance between synthetic and treated units

|  | Treated | Synthetic | Sample Mean |
| --- | --- | --- | --- |
| special.pRate.1990 | 68.000 | 67.005 | 34.180 |
| special.pRate.1991.1995 | 60.620 | 62.152 | 30.590 |
| special.pRate.1996.1998 | 63.633 | 60.236 | 28.987 |

#### Placebo testing by country and time


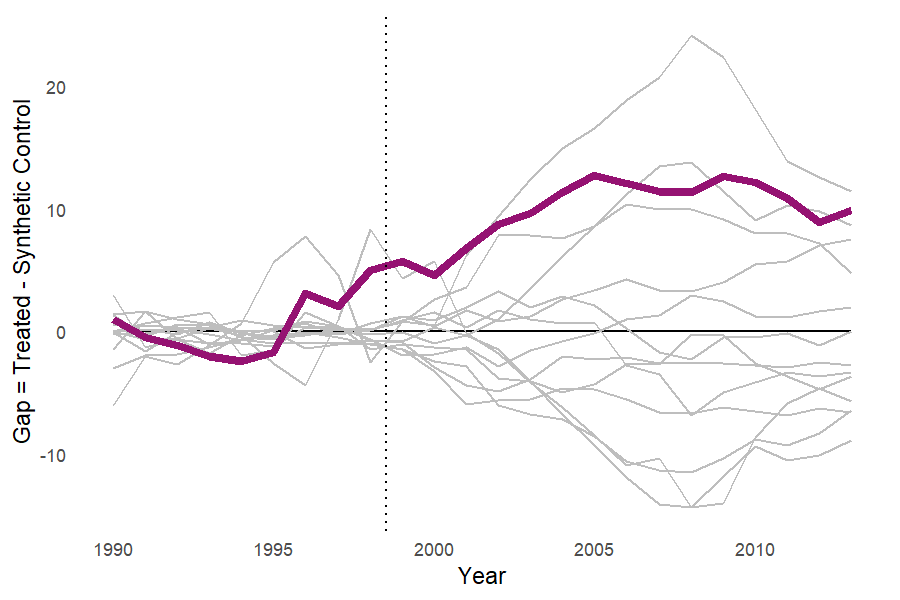


In placebo-country tests, England and Wales ranked 9 out of 15 countries by pre/post-MSPE ratio.


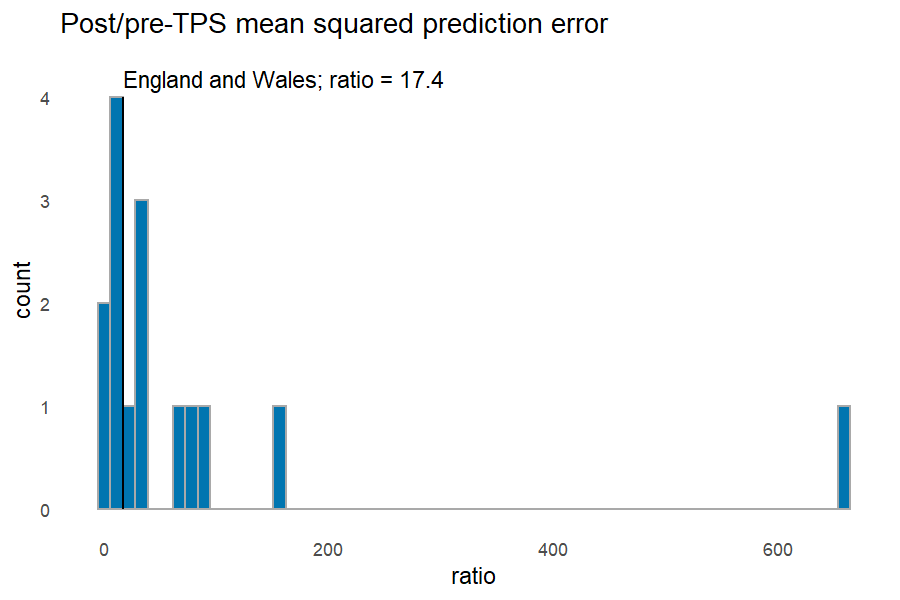


## Adding all predictors

### Model 5: Under-18 birth rates

#### England vs Synthetic Control


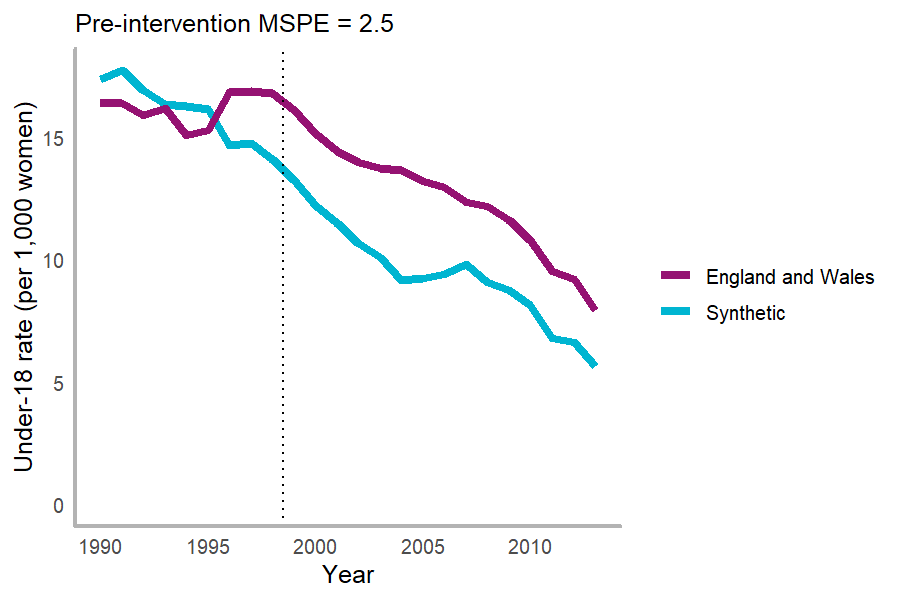


Loss-V (For optimised period) = 2.5, MSPE (for whole pre-intervention observation period) = 2.5.

#### Weights and balance

Country weights

| Country | Weight |
| --- | --- |
| Italy | 0.374 |
| Iceland | 0.328 |
| United States of America | 0.298 |
| Switzerland | 0.000 |
| Germany | 0.000 |
| Denmark | 0.000 |
| Spain | 0.000 |
| Finland | 0.000 |
| France | 0.000 |
| Netherlands | 0.000 |
| Norway | 0.000 |
| Portugal | 0.000 |
| Sweden | 0.000 |

Predictor weights

|  | v.weights |
| --- | --- |
| special.rate.1990.1993 | 0.028 |
| special.rate.1994 | 0.023 |
| special.rate.1995 | 0.034 |
| special.rate.1996.1998 | 0.15 |
| special.GDPperCap.1990.2003 | 0.014 |
| special.GDPperCap.2004 | 0.078 |
| special.GDPperCap.2005.2010 | 0.014 |
| special.GDPperCap.2011.2013 | 0.002 |
| special.edu_spend.1995.1998 | 0.003 |
| special.edu_spend.1999.2007 | 0 |
| special.edu_spend.2008.2013 | 0.13 |
| special.MobilePhones.1990.1992 | 0 |
| special.MobilePhones.1993.1995 | 0.022 |
| special.MobilePhones.1996.1997 | 0.011 |
| special.MobilePhones.1998.2013 | 0.126 |
| special.UrbanPop.1990 | 0.092 |
| special.UrbanPop.1991 | 0.088 |
| special.UrbanPop.1992 | 0.087 |
| special.UrbanPop.1993.2013 | 0.098 |

Predictor balance between synthetic and treated units

|  | Treated | Synthetic | Sample Mean |
| --- | --- | --- | --- |
| special.rate.1990.1993 | 16.214 | 17.099 | 8.005 |
| special.rate.1994 | 15.078 | 16.268 | 7.329 |
| special.rate.1995 | 15.285 | 16.157 | 7.099 |
| special.rate.1996.1998 | 16.836 | 14.511 | 6.703 |
| special.GDPperCap.1990.2003 | 24800.333 | 26808.216 | 26870.008 |
| special.GDPperCap.2004 | 39983.985 | 39557.010 | 39108.220 |
| special.GDPperCap.2005.2010 | 43340.344 | 44960.799 | 47573.339 |
| special.GDPperCap.2011.2013 | 41975.732 | 43998.534 | 52420.115 |
| special.edu_spend.1995.1998 | 4.223 | 5.330 | 5.616 |
| special.edu_spend.1999.2007 | 5.296 | 5.885 | 5.627 |
| special.edu_spend.2008.2013 | 5.794 | 5.884 | 5.691 |
| special.MobilePhones.1990.1992 | 2.256 | 2.896 | 2.778 |
| special.MobilePhones.1993.1995 | 6.881 | 7.278 | 7.214 |
| special.MobilePhones.1996.1997 | 13.808 | 18.177 | 18.310 |
| special.MobilePhones.1998.2013 | 98.113 | 95.733 | 93.736 |
| special.UrbanPop.1990 | 78.140 | 77.151 | 74.238 |
| special.UrbanPop.1991 | 78.112 | 77.323 | 74.478 |
| special.UrbanPop.1992 | 78.172 | 77.514 | 74.734 |
| special.UrbanPop.1993.2013 | 79.690 | 79.327 | 77.582 |

#### Placebo testing by country


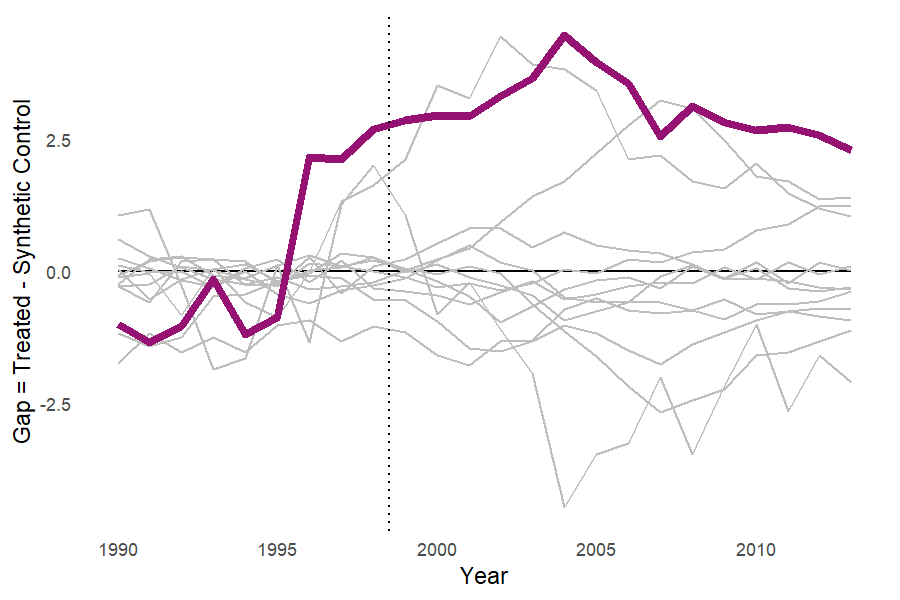


In placebo-country tests, England and Wales ranked 5 out of 14 countries by pre/post-MSPE ratio.


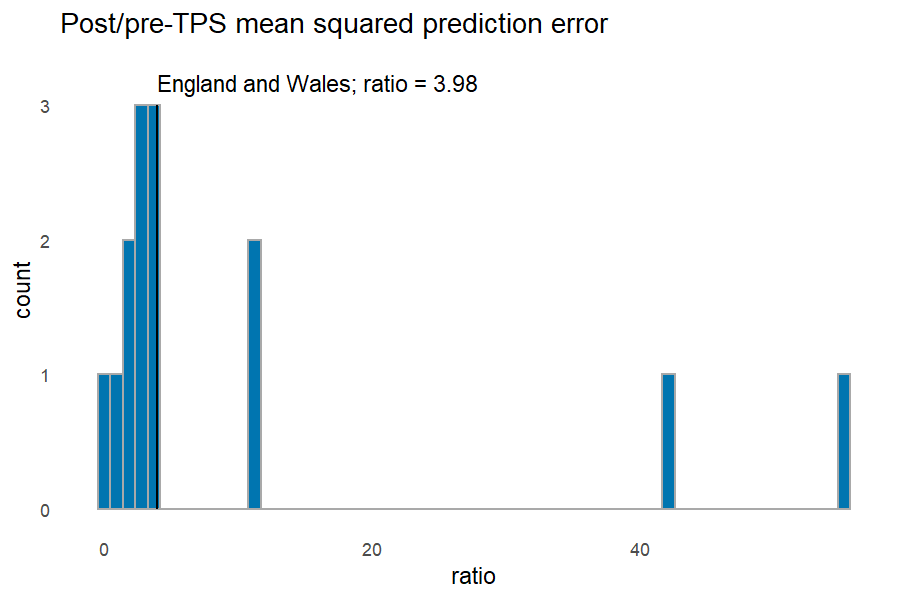


### Model 6: Under-20 pregnancy rates

#### England vs Synthetic Control


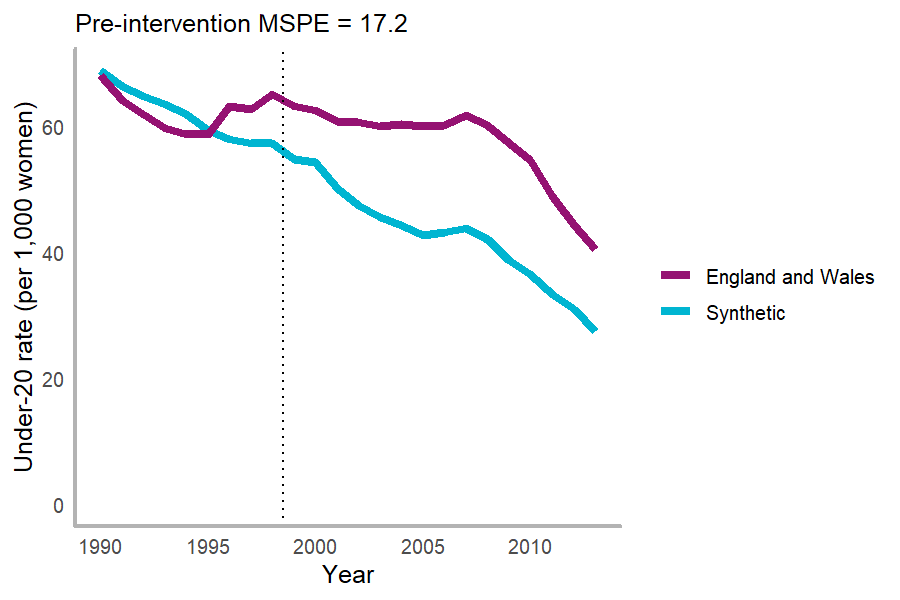


Loss-V (For optimised period) = 17.2, MSPE (for whole pre-intervention observation period) = 17.2.

#### Weights and balance

Country weights

| Country | Weight |
| --- | --- |
| United States of America | 0.522 |
| Iceland | 0.278 |
| Italy | 0.201 |
| Switzerland | 0.000 |
| Germany | 0.000 |
| Denmark | 0.000 |
| Spain | 0.000 |
| Finland | 0.000 |
| France | 0.000 |
| Netherlands | 0.000 |
| Norway | 0.000 |
| Portugal | 0.000 |
| Sweden | 0.000 |

Predictor weights

|  | v.weights |
| --- | --- |
| special.pRate.1990 | 0.175 |
| special.pRate.1991.1995 | 0.156 |
| special.pRate.1996.1998 | 0.315 |
| special.MobilePhones.1990.1992 | 0 |
| special.MobilePhones.1993.1995 | 0.001 |
| special.MobilePhones.1996.1997 | 0.004 |
| special.MobilePhones.1998.2013 | 0.054 |
| special.edu_spend.1995.1998 | 0 |
| special.edu_spend.1999.2007 | 0 |
| special.edu_spend.2008.2013 | 0.001 |
| special.GDPperCap.1990.2003 | 0 |
| special.GDPperCap.2004 | 0.014 |
| special.GDPperCap.2005.2010 | 0 |
| special.GDPperCap.2011.2013 | 0.006 |
| special.UrbanPop.1990 | 0.11 |
| special.UrbanPop.1991 | 0.087 |
| special.UrbanPop.1992 | 0.071 |
| special.UrbanPop.1993.2013 | 0.004 |

Predictor balance between synthetic and treated units

|  | Treated | Synthetic | Sample Mean |
| --- | --- | --- | --- |
| special.pRate.1990 | 68.000 | 68.849 | 32.961 |
| special.pRate.1991.1995 | 60.620 | 63.155 | 29.104 |
| special.pRate.1996.1998 | 63.633 | 57.510 | 27.083 |
| special.MobilePhones.1990.1992 | 2.256 | 3.182 | 2.778 |
| special.MobilePhones.1993.1995 | 6.881 | 8.184 | 7.214 |
| special.MobilePhones.1996.1997 | 13.808 | 18.494 | 18.310 |
| special.MobilePhones.1998.2013 | 98.113 | 85.702 | 93.736 |
| special.edu_spend.1995.1998 | 4.223 | 5.570 | 5.616 |
| special.edu_spend.1999.2007 | 5.296 | 6.133 | 5.627 |
| special.edu_spend.2008.2013 | 5.794 | 6.239 | 5.691 |
| special.GDPperCap.1990.2003 | 24800.333 | 28604.113 | 26870.008 |
| special.GDPperCap.2004 | 39983.985 | 41170.217 | 39108.220 |
| special.GDPperCap.2005.2010 | 43340.344 | 46569.676 | 47573.339 |
| special.GDPperCap.2011.2013 | 41975.732 | 46884.569 | 52420.115 |
| special.UrbanPop.1990 | 78.140 | 77.870 | 74.238 |
| special.UrbanPop.1991 | 78.112 | 78.125 | 74.478 |
| special.UrbanPop.1992 | 78.172 | 78.389 | 74.734 |
| special.UrbanPop.1993.2013 | 79.690 | 80.692 | 77.582 |

#### Placebo testing by country


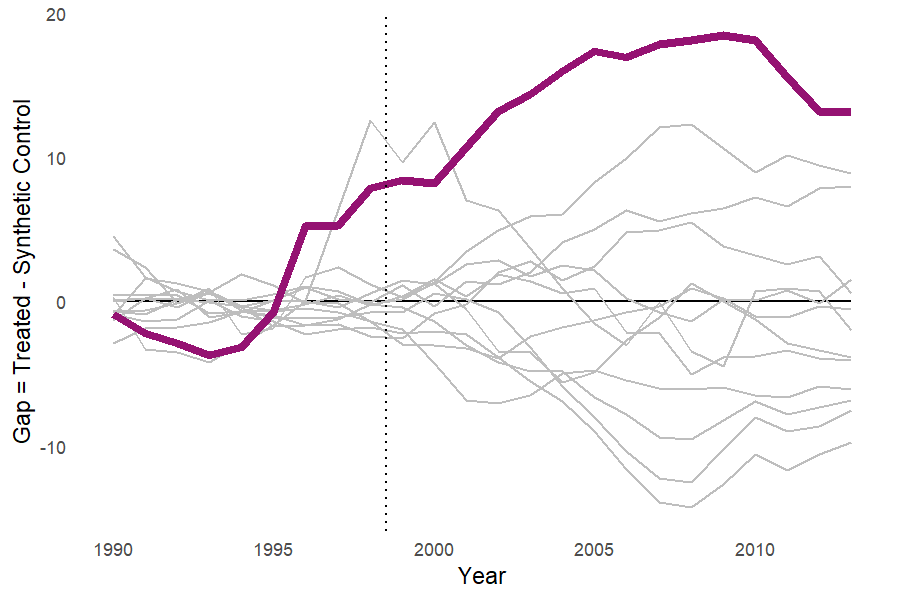


In placebo-country tests, England and Wales ranked 8 out of 14 countries by pre/post-MSPE ratio.


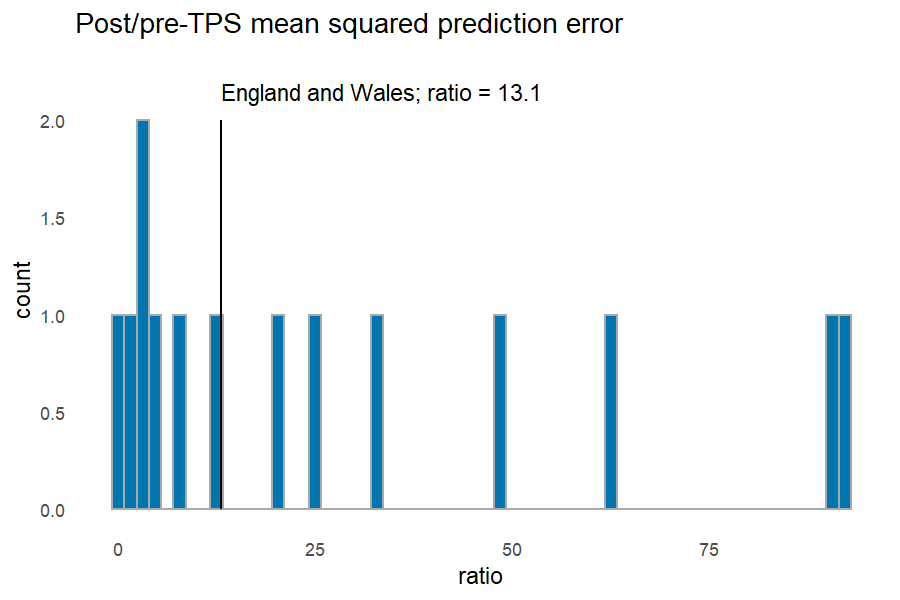


# Model-testing iterations

## Under-18 pregnancy - no added predictors

Plotting MSPEs by iteration and gaps for all iterations.


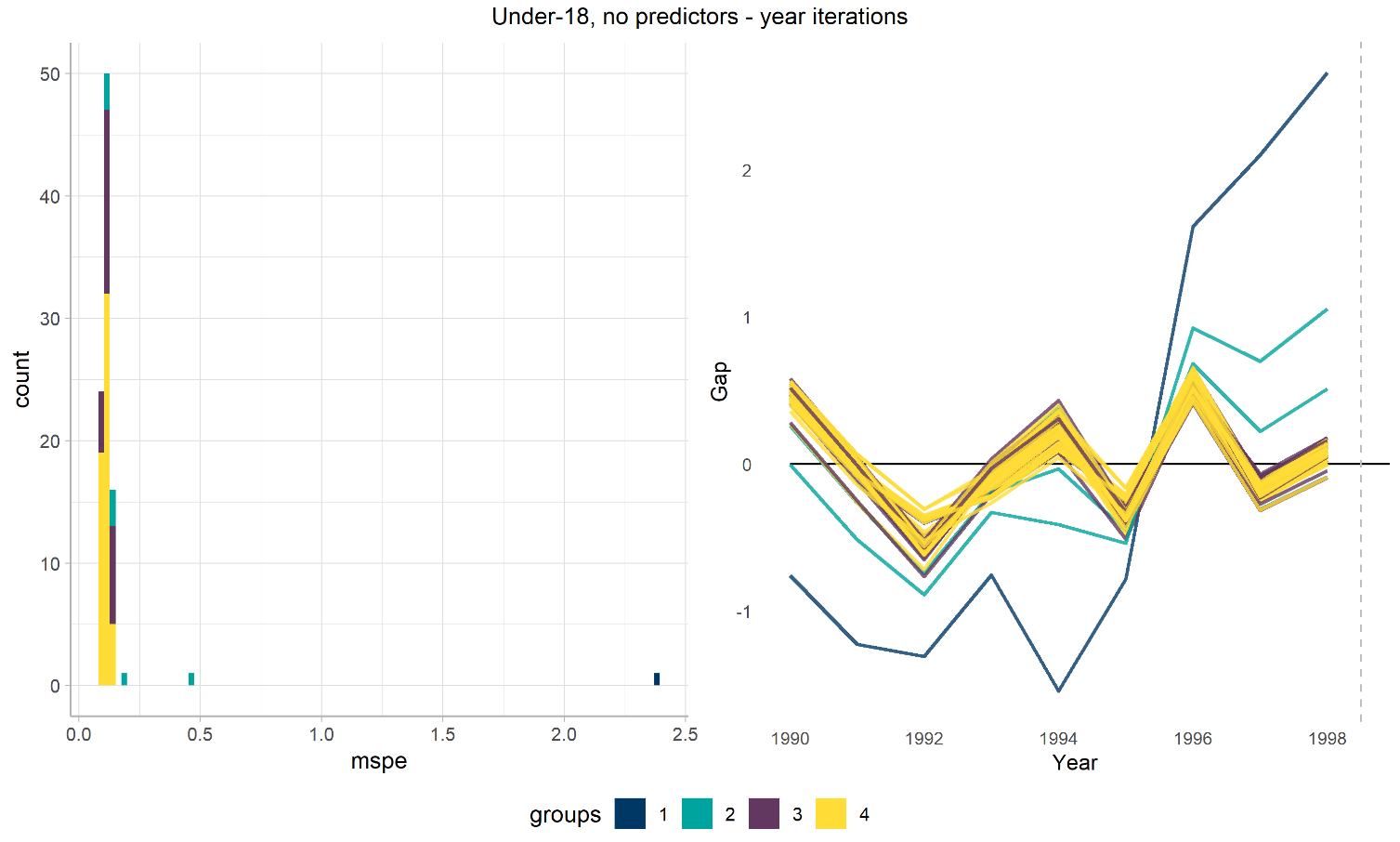


### Top countries

Differing iterations produced different country weightings. I exctracted the top country for each iteration and plotted the lowest MSPE for the top four countries:


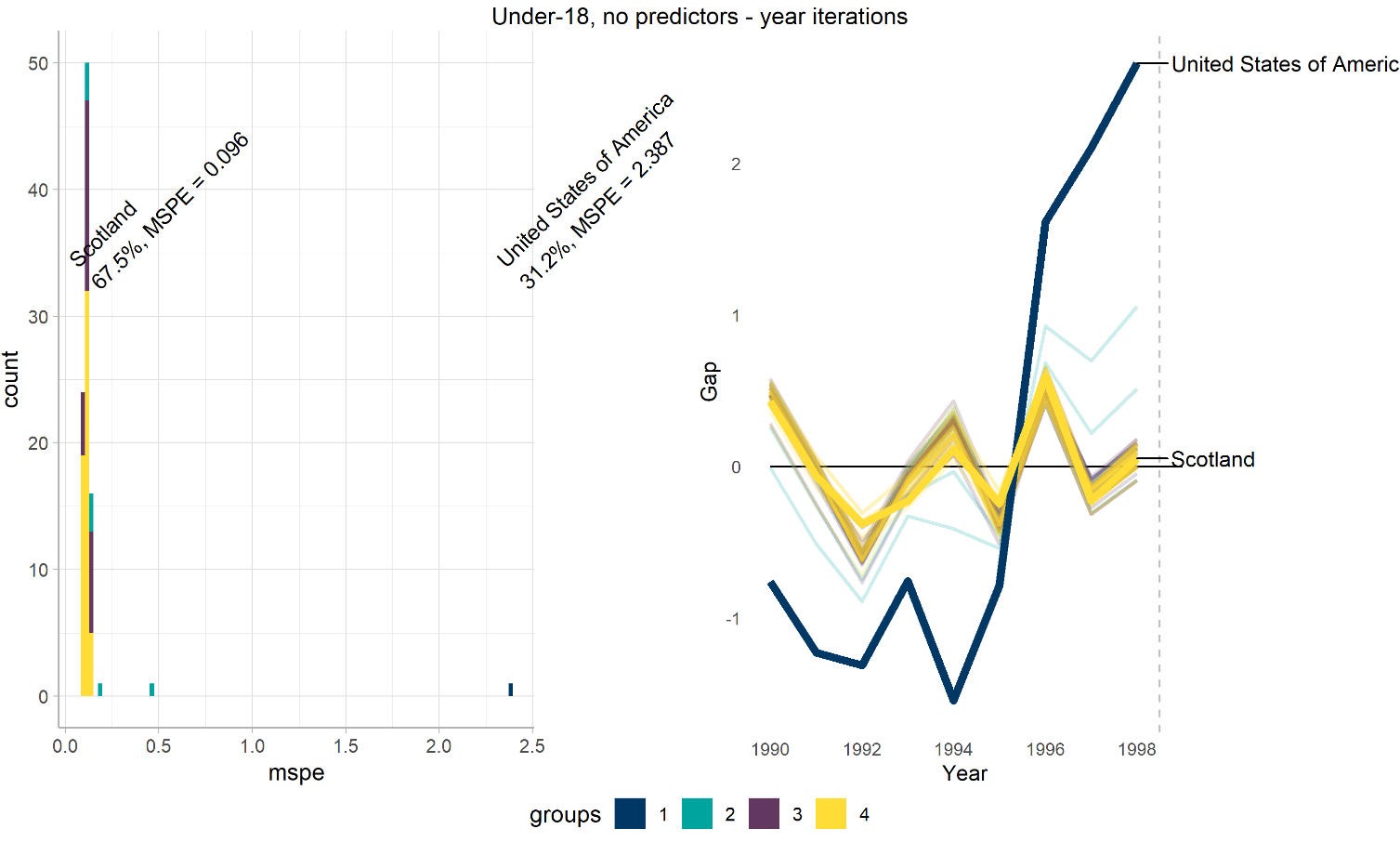


### Removing top countries

A further iterative cycle constructed sequential synthetic controls, removing the previous top-weighted country to test for over-reliance on very few abnormal countries. Labels represent the top-weighted country in each analysis by weight and pre-intervention MSPE:


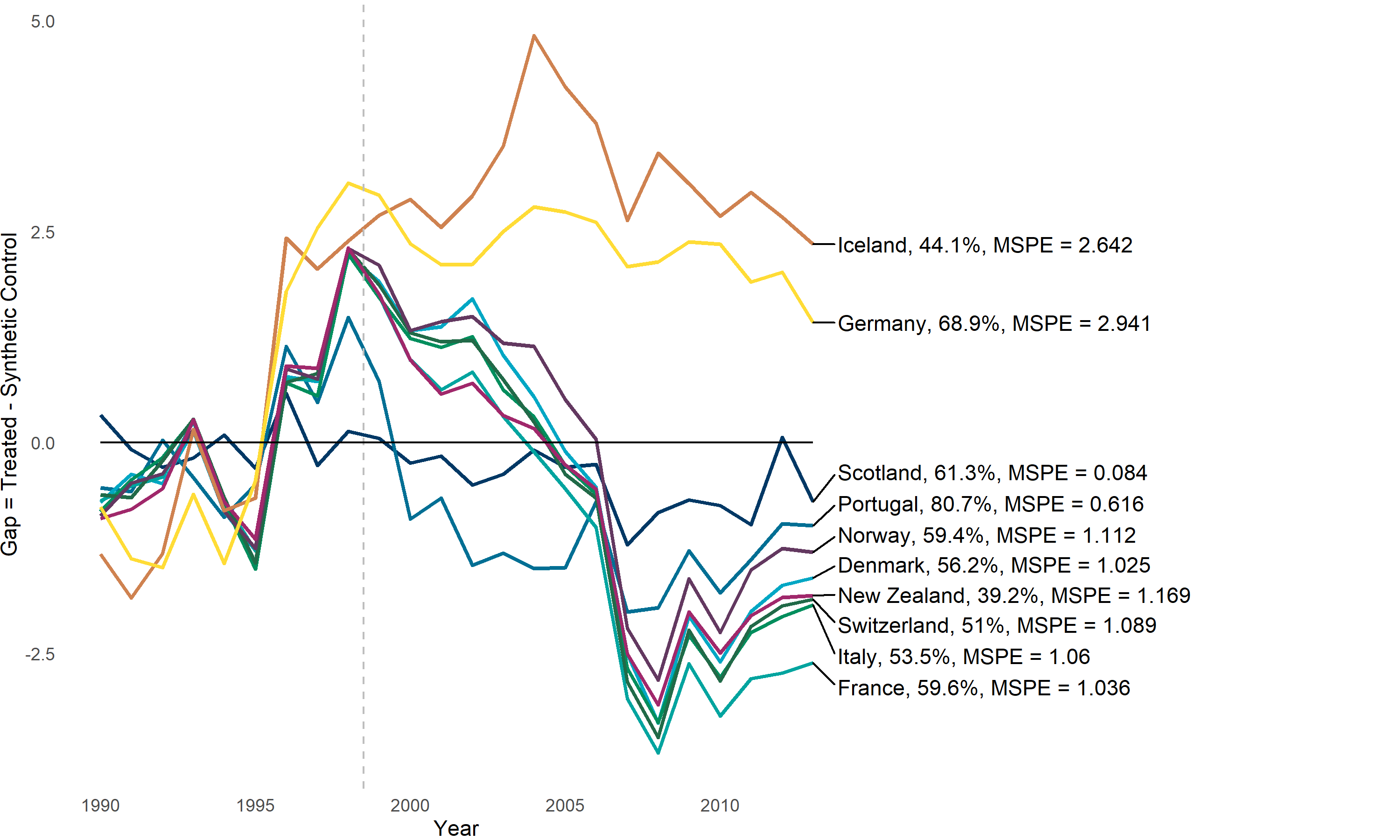


## Under-20 - no added predictors


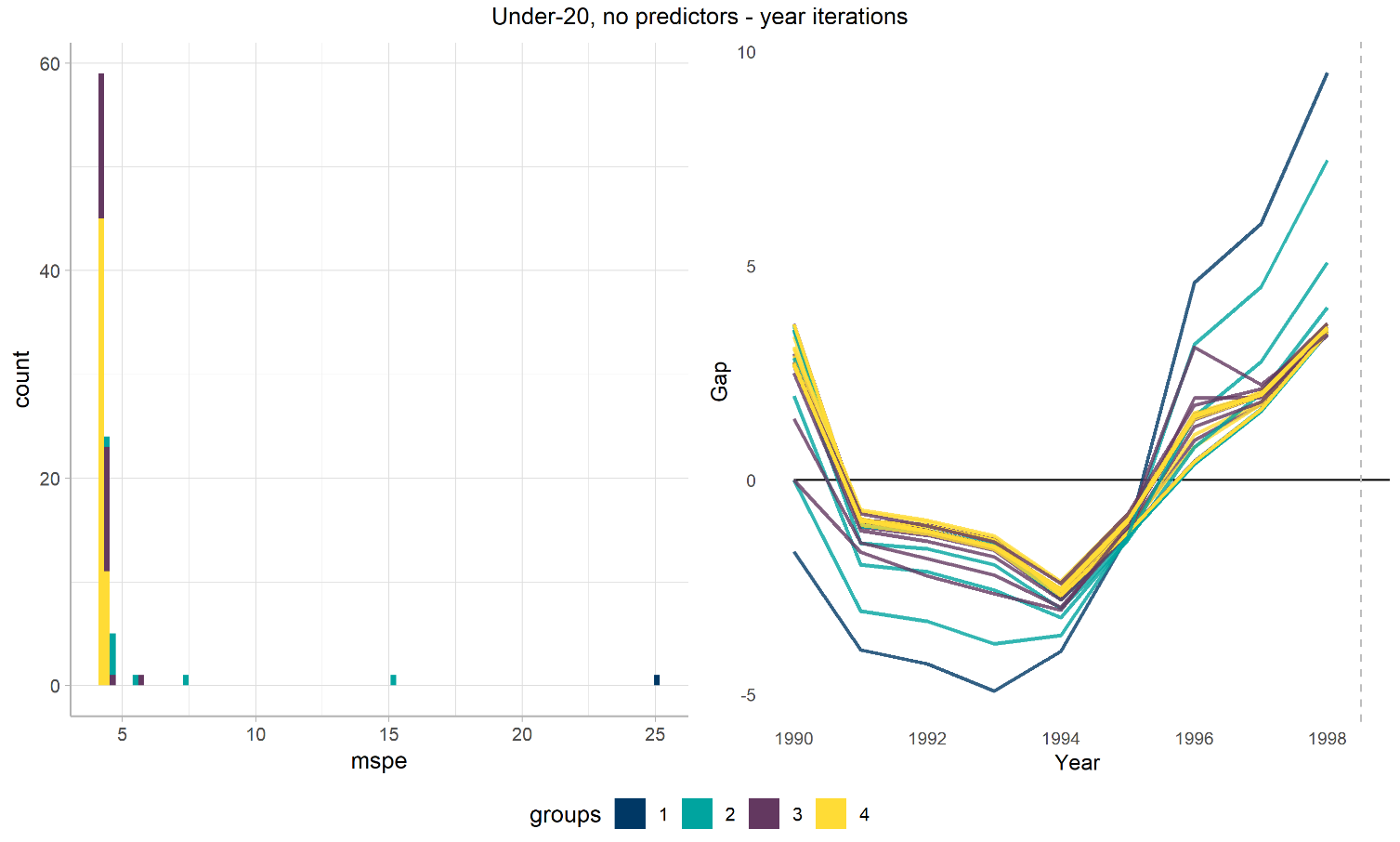


### Top Countries


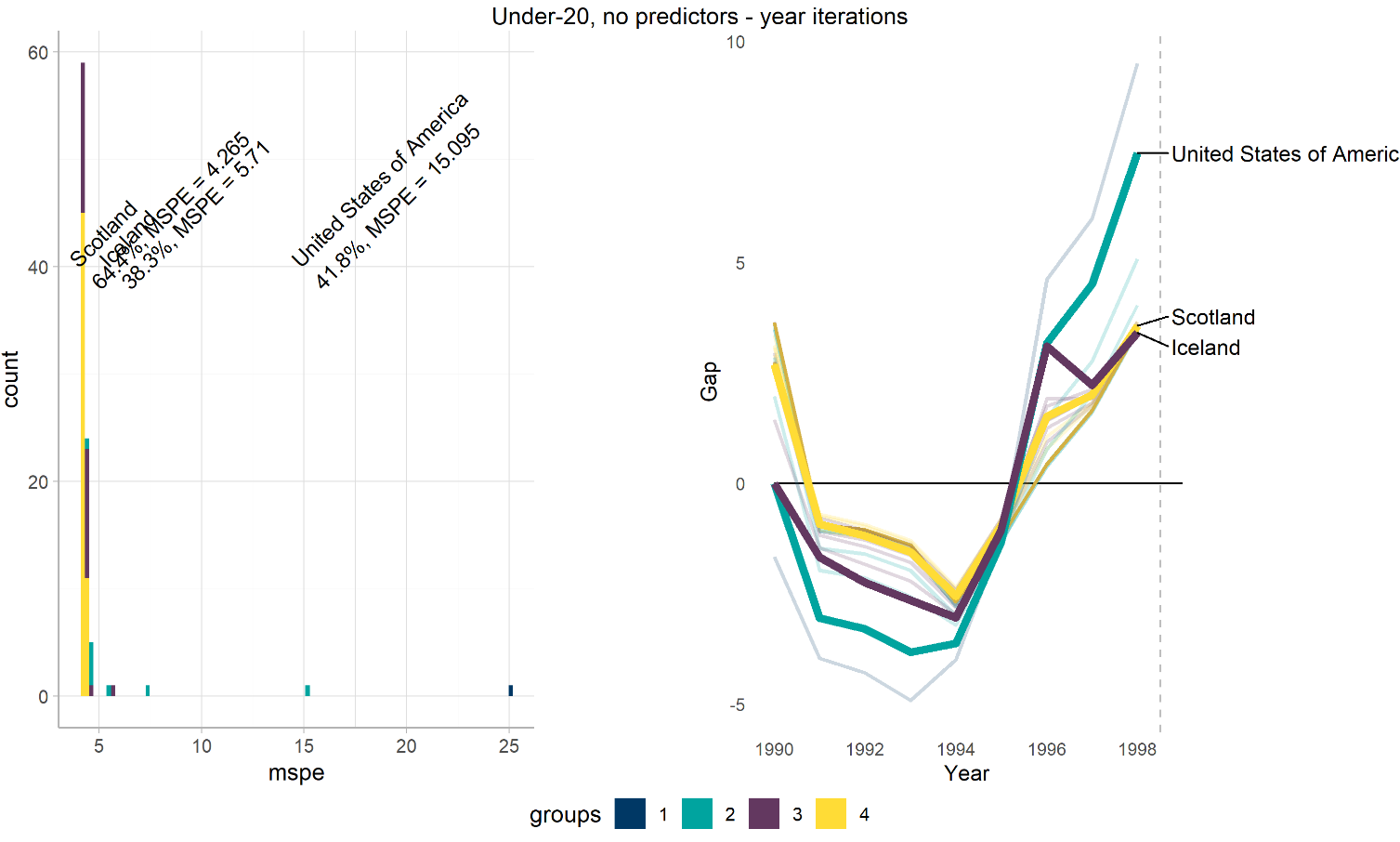


### Removing Top Countries


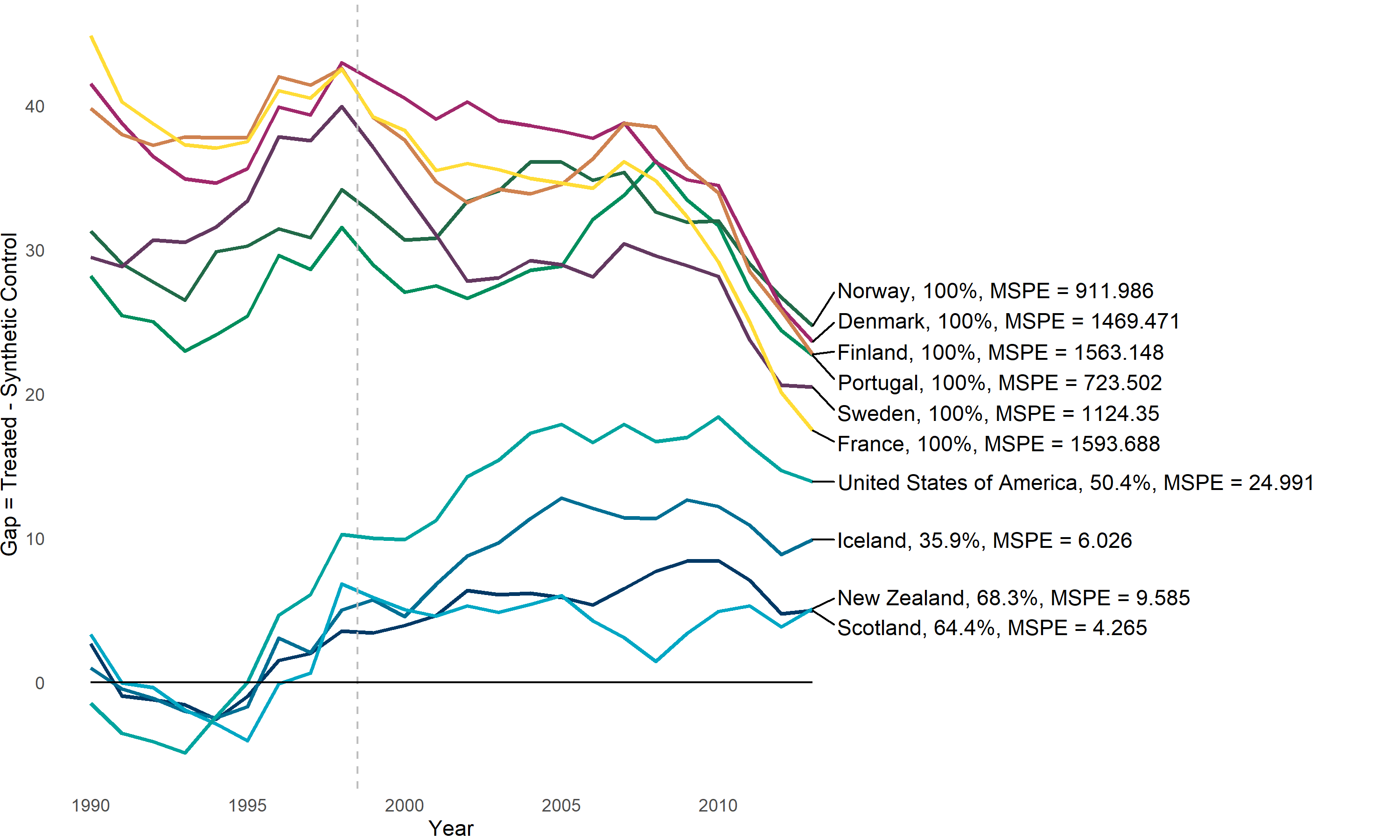

Supplement: Multimedia component 1 [file mmc1.docx]
